# Calcium regulates the mycophagous ability of *Burkholderia gladioli* strain NGJ1 in a type III secretion system-dependent manner

**DOI:** 10.1186/s12866-020-01897-2

**Published:** 2020-07-20

**Authors:** Sunil Kumar Yadav, Joyati Das, Rahul Kumar, Gopaljee Jha

**Affiliations:** Plant Microbe Interactions Laboratory, National Institute of Plant Genome Research, Aruna Asaf Ali Marg, New Delhi, 110067 India

**Keywords:** Mycophagy, Bacterial-fungal interaction, T3SS, Effectors, *Endo-β-1*, *3- glucanase*

## Abstract

**Background:**

A rice associated bacterium *Burkholderia gladioli* strain NGJ1 demonstrates mycophagy, a phenomenon wherein bacteria feed on fungi. Previously, we have reported that NGJ1 utilizes type III secretion system (T3SS) to deliver a prophage tail-like protein (Bg_9562) into fungal cells to establish mycophagy.

**Results:**

In this study, we report that calcium ion concentration influences the mycophagous ability of NGJ1 on *Rhizoctonia solani*, an important fungal pathogen. The calcium limiting condition promotes mycophagy while high calcium environment prevents it. The expression of various T3SS apparatus encoding genes of NGJ1 was induced and secretion of several potential T3SS effector proteins (including Bg_9562) into extracellular milieu was triggered under calcium limiting condition. Using LC-MS/MS proteome analysis, we identified several calcium regulated T3SS effector proteins of NGJ1. The expression of genes encoding some of these effector proteins was upregulated during mycophagous interaction of NGJ1 with *R. solani*. Further, mutation of one of these genes (endo-β-1, 3- glucanase) rendered the mutant NGJ1 bacterium defective in mycophagy while complementation with full length copy of the gene restored its mycophagous activity.

**Conclusion:**

Our study provides evidence that low calcium environment triggers secretion of various T3SS effectors proteins into the extracellular milieu and suggests the importance of cocktail of these proteins in promoting mycophagy.

## Background

Bacteria are simplest living, single-celled, micro-organisms and are omnipresent in diverse natural habitats like soil, water, air, etc. They are also found in association with or inhabiting within multicellular organisms and demonstrate diverse interactions such as mutualism, commensalism, antagonism or parasitism with other co-habiting organisms [1, 2]. Bacteria which demonstrate antagonistic interactions with fungi are considered as potential biocontrol agents [3–6]. Interestingly amongst the anti-fungal bacteria, a few have been reported to grow and multiply at the cost of living fungal biomass. Such a strategy wherein bacteria exploits fungi as a source of nutrients is known as bacterial mycophagy [7]. One of the first direct evidence of bacterial mycophagy has been reported in *Collimonas* sp. which can utilize fungal hyphae as an energy source [8, 9]. Similarly, *Staphylococcus aureus* can feed on *Cryptococcus* by inducing cell death response and *Aeromonas caviae* can feed on *Rhizoctonia solani* as well as *Fusarium oxysporum* [7, 10, 11]*.* Also, *Burkholderia terrae* can feed on a soil fungus (*Lyophyllum* sp. strain Karsten); potentially by utilizing glycerol from the fungi as an energy source [12]. Notably various genes related to chemotaxis, motility, stress response, energy metabolism etc. are found upregulated during *B. terrae- Lyophyllum* sp. interactions [13]. Although there are many reports of mycophagous bacteria, the mechanistic insight about bacterial mycophagy remains largely unknown.

In our previous study, we reported a novel bacterium *Burkholderia gladioli* strain NGJ1 which demonstrates mycophagous activity against various fungal species including *Rhizoctonia solani* [14]. Upon confrontation with *R. solani*, NGJ1 damages fungal hyphae by inducing cell death and potentially utilizes the fungal biomass to promote its own growth. It was also demonstrated that NGJ1 utilizes a prophage tail-like protein (Bg_9562) as a type III secretion system (T3SS) effector to feed on fungi. The T3SS is a tiny injection like nano-machine present in gram-negative bacteria which is used to deliver effector proteins directly into the host cell in a contact-dependent manner [15–17]. The presence of various metal ions especially calcium and iron have been reported to control T3SS in various bacteria [18–21]. High calcium condition inhibits the assembly of T3SS apparatus and prevents secretion of effector proteins. Whereas low calcium condition induces the formation of T3SS apparatus and promotes T3SS mediated secretion of effector proteins [19, 22–26]. Various reports suggest that low calcium condition can be created in laboratory media by supplementation of a high-affinity calcium chelator, EGTA (ethylene glycol-bis (β-amino ethyl ether)-N, N, N′, N′-tetra acetic acid) [27–30]. The EGTA mediated low calcium environment is known to trigger secretion of T3SS effectors (into extracellular milieu) in many gram-negative bacteria such as *Pseudomonas aeruginosa*, *Yersinia* sp., *Escherichia coli*, *Vibrio parahaemolyticus, Aeromonas hydrophila,* etc. [19, 24, 30–35]. It has been anticipated that EGTA mediated low calcium environment can mimic host cell contact and stimulates the secretion of T3SS effectors into extracellular milieu [36]. During pathogenesis, bacteria may sense calcium limiting condition in the host which stimulates secretion of various T3SS effectors [37–39].

The importance of T3SS and its effector proteins during interaction of plant and animal pathogenic bacteria with their respective hosts have been widely described [17, 18, 40]. However, involvement of T3SS and its effectors during bacterial-fungal interaction remain largely elusive. The role of T3SS in altering the physiology of co-habiting fungi and influencing their ecological fitness had been described [41]. Also the involvement of bacterial T3SS during symbiotic bacterial-fungal interactions has been proposed. For example, the T3SS of *B. rhizoxinica* has been found to promote sporulation of its associated fungi, *Rhizopus microsporus* [42]. In the present study, we demonstrate that EGTA mediated low calcium condition promotes mycophagous ability of NGJ1 during bacterial-fungal confrontation in a T3SS dependent manner. We have identified various potential T3SS effectors (including Bg_9562) that are secreted by NGJ1 when grown under low calcium condition. Using mutation and complementation studies, we demonstrate the importance of one of these T3SS effector (endo- β-1, 3- glucanase) proteins in bacterial mycophagy.

## Results

### Calcium inhibits mycophagous activity of NGJ1

**Fig. 1:**
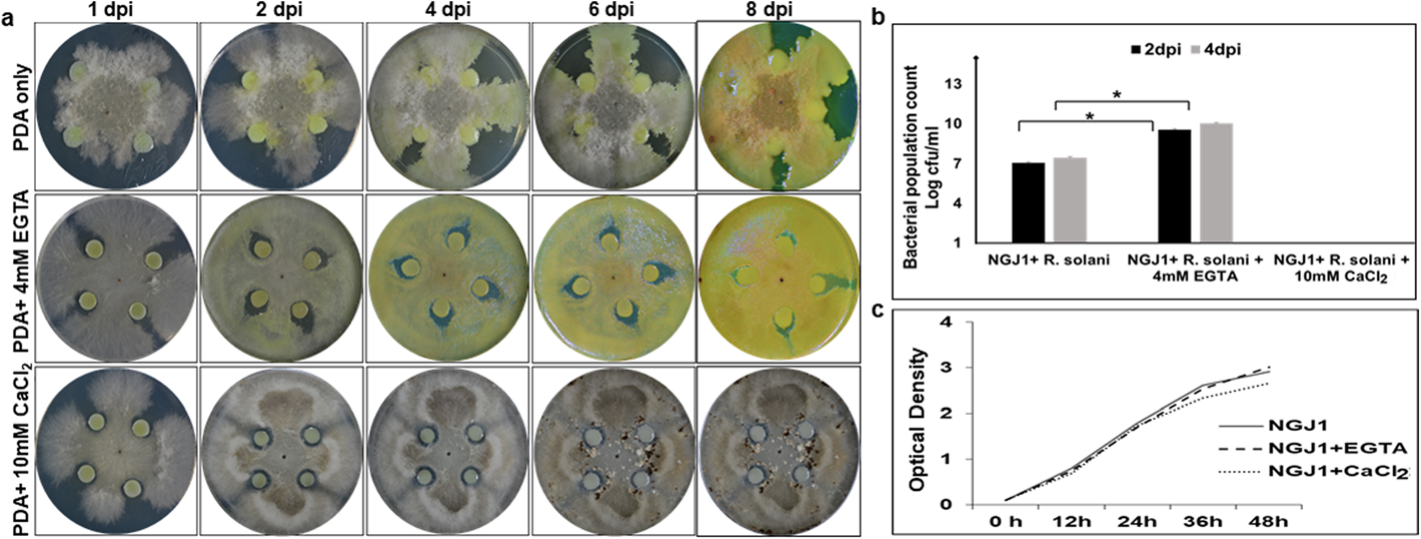
Low calcium condition promotes mycophagous activity of NGJ1**. a** Mycophagous activity of NGJ1 on *R. solani* on PDA plates with or without EGTA/CaCl_2_ supplementation. On EGTA supplemented plates, NGJ1 grew over the mycelial mass in less than 4 days while on PDA plates (without supplementation), the bacterium took 7–9 days to cover the fungal biomass. Supplementation of CaCl_2_ completely inhibited mycophagous ability and prevented bacterial growth over fungal biomass. **b** The abundance of NGJ1 on *R. solani* mycelia on confrontation plates supplemented with or without EGTA/CaCl_2_. The bacterial abundance was significantly more on EGTA containing PDA plates than that on PDA only or CaCl_2_ supplemented plates. **c** The NGJ1 growth pattern in PDB with or without EGTA/CaCl_2_ supplementation. The growth was similar under tested conditions. Similar results were obtained in at least three independent biological experiments and only representative photographs are shown. Asterisk * indicates significant difference at *P* < 0.05 (estimated using one-way ANOVA). Graphs show mean values ± standard deviation

Divalent cationic salts are involved in various cellular and molecular processes of living micro-organisms. We analyzed the effect of some divalent cationic salts (MgCl_2_, CaCl_2,_ and FeCl_2_) on mycophagous ability of *Burkholderia gladioli* strain NGJ1. Upon confrontation with *Rhizoctonia solani* on PDA plates, NGJ1 demonstrates mycophagy by spreading over fungal biomass. The supplementation of 4 mM and 10 mM CaCl_2_ inhibited NGJ1 to forage over *R. solani* mycelia (Fig. 1, Additional file: Fig. S1 and Table S1). However, the supplementation of MgCl_2_ (1, 4 and 10 mM) and FeCl_2_ (1, 4 and 10 mM) had no apparent effect on mycophagous ability of NGJ1 on *R. solani* (Additional file: Fig. S1 and Table S1). Upon bacterial confrontation, hundreds of viable secondary sclerotia (the spore-like resting structure) of *R. solani* were produced on CaCl_2_ supplemented plates (Additional file: Tables S2 and S3). On the other hand, only a few non-viable secondary sclerotia were produced on PDA plates without CaCl_2_ supplementation. Similarly, on MgCl_2_/ FeCl_2_ supplemented PDA plates only a few non-viable secondary sclerotia were observed.

We further investigated the effect of CaCl_2_ supplementation on mycophagous ability of NGJ1 on a semi-synthetic minimal media i.e. CDA (Czapek Dox Agar) that lacks calcium. Previously, we had reported that NGJ1 demonstrates enhanced mycophagy on CDA, in comparison to that on PDA [14]. In this study, we observed gradual reduction in the mycophagous ability of NGJ1 with increase in concentration of CaCl_2_ supplementation in CDA (Additional file: Fig. S2A). The bacterial growth in presence of *R. solani* mycelia was significantly less in CaCl_2_ supplemented CDB (Czapek Dox Broth) media than that observed in only CDB (Additional file: Fig. S2B). Taken together, these results suggested that presence of high concentration of CaCl_2_ during bacterial-fungal confrontation is inhibitory for bacterial mycophagy. Here it is noteworthy that CaCl_2_ supplementation had no adverse effect on the growth of NGJ1 and *R. solani* (Fig. 1c, Additional file: Fig. S2B).

### Low calcium condition promotes the mycophagous ability of NGJ1

Using ICP-MS (Inductively Coupled Plasma Mass Spectrometry) we estimated the calcium ion concentration in confrontation media during NGJ1-*R. solani* interaction. The PDA, PDB as well as the cell-free culture supernatants of bacteria (NGJ1) or fungi (*R. solani*) were found to contain > 200 ppb concentration of calcium ions (Additional file: Table S4). However, during 24 h of bacterial-fungal confrontation, calcium ion concentration in the culture supernatant was reduced to < 200 ppb. It was further reduced to < 100 ppb during 48 h of confrontation (Additional file: Table S4). Overall, the ICP-MS analysis suggested that a low calcium environment is created during bacterial mycophagy.

To further study the effect of low calcium on mycophagous ability of NGJ1, we created a calcium limiting condition in confrontation media by supplementation of various concentrations (1, 4 and 10 mM) of EGTA, a high-affinity calcium ion chelator. The presence of 4 mM but not 1 mM EGTA stimulated the mycophagous activity, while 10 mM EGTA was inhibitory for the growth of bacteria as well as fungi (Fig. 1a, Additional file: Fig. S1 and Table S1). Generally, NGJ1 spreads over fungal biomass in 6–9 days of confrontation; however in presence of 4 mM EGTA it covered the fungal biomass in only 3–4 days (Fig. 1a). Also, the bacterial abundance on EGTA supplemented confrontation plates was significantly higher compared to that observed on control (without EGTA) plates (Fig. 1b). The number of secondary sclerotia produced on EGTA supplemented plates was limited to a few (Additional file: Table S2) and mostly they were unable to germinate when transferred on to fresh PDA plates (Additional file: Table S3).

Besides EGTA, we also tested the effect of other cationic chelators (EDTA and dipyridyl) on mycophagous ability of NGJ1. The supplementation of EDTA or dipyridyl in confrontation plates had no apparent effect on the ability of NGJ1 to forage over *R. solani* mycelia (Additional file: Fig. S1 and Table S1). After confrontation, only a limited number of secondary sclerotia were produced on these plates (Additional file: Table S2) and they were mostly non-viable (Additional file: Table S3)**.** It is noteworthy that supplementation of 4 mM concentration of EGTA/EDTA/dipyridyl had no apparent adverse effect on the growth of NGJ1 and *R. solani* (Fig. 1c and Additional file: Fig. S1).

### Calcium regulates the expression of T3SS apparatus encoding genes

**Fig. 2:**
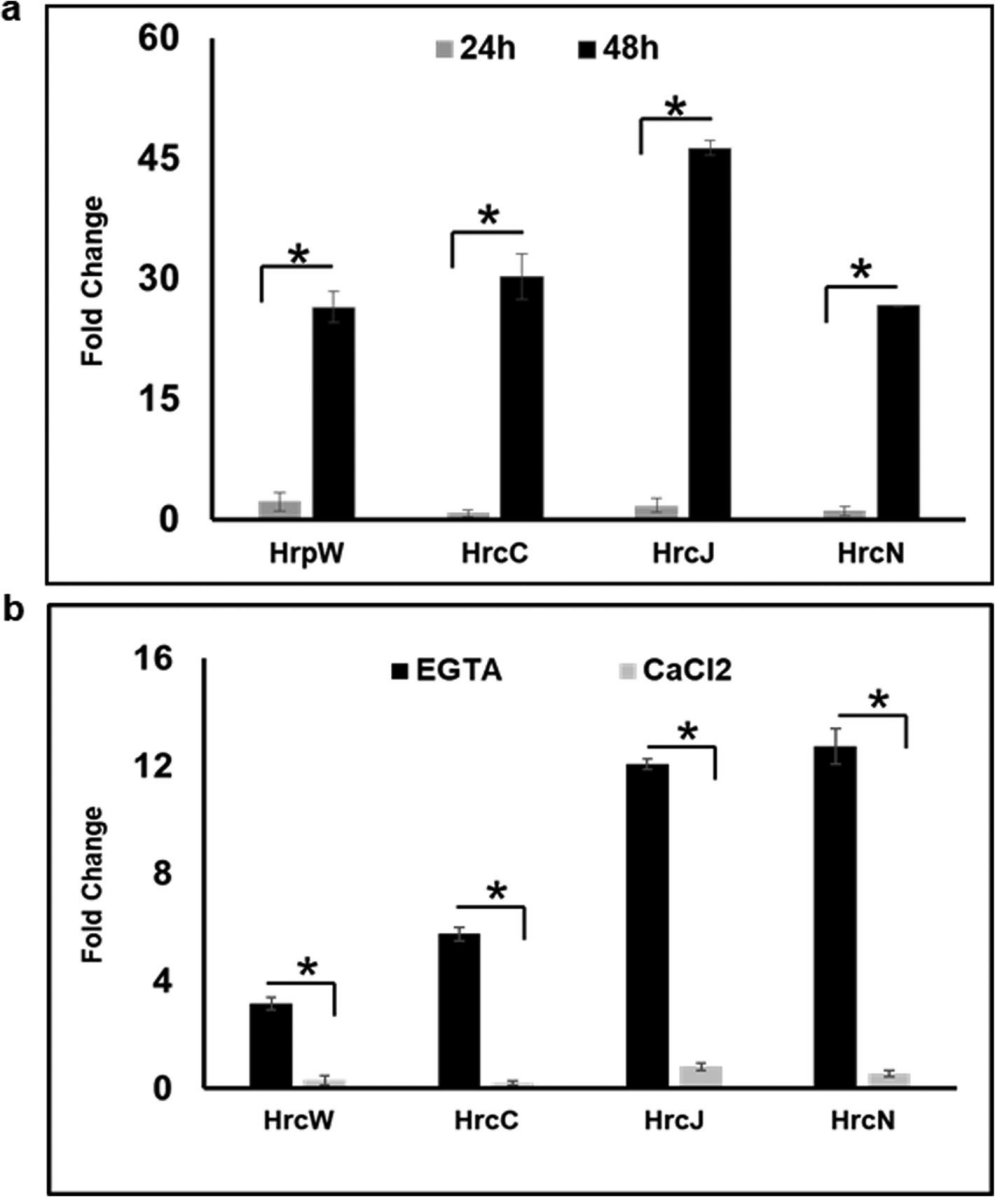
Expression dynamics of T3SS apparatus encoding genes of NGJ1. **a** RT-PCR based expression analysis of T3SS apparatus encoding genes of NGJ1 during mycophagous interaction with *R. solani* in PDB. The differential gene expression was estimated during mycophagy with respect to the expression observed during bacterial growth in absence of fungi. **b** RT-PCR based expression analysis of T3SS apparatus encoding genes of NGJ1 in PDB with or without EGTA/CaCl_2_ supplementation. The differential gene expression was calculated in presence of EGTA/CaCl_2_ with respect to that observed in absence of EGTA/CaCl_2._ The 16S rRNA gene of NGJ1 was used as endogenous control in RT-PCR analysis. The experiments were independently repeated three times with a minimum three technical replicates. Asterisks * indicate significant difference at *P* < 0.05 (estimated using one-way ANOVA). Graphs show mean values ± standard deviation

We have previously reported that a T3SS mutant strain (NGJ12) of NGJ1 is defective in mycophagy [14]. We tested whether calcium limiting condition may restore the mycophagous ability in NGJ12 and observed that even in presence of EGTA, the NGJ12 is unable to exhibit mycophagy on *R. solani* (Additional file: Fig. S3). Considering this, we anticipated that a functional T3SS might be required for low calcium condition triggered mycophagous ability of NGJ1. Notably, most of the T3SS apparatus encoding genes were > 10 fold upregulated during 48 h of mycophagous interaction of NGJ1 with *R. solani*, compared to that observed during NGJ1 growth in PDB without *R. solani* (Fig. 2a). However, supplementation of CaCl_2_ (10 and 20 mM) significantly decreased the expression of these genes during 48 h of mycophagous interaction of NGJ1 with *R. solani* (Additional file: Fig. S4).

Further, we analyzed the expression of T3SS apparatus encoding genes during NGJ1 growth in PDB with or without EGTA/CaCl_2_ supplementation (in absence of *R. solani*). EGTA induced the expression of T3SS apparatus encoding genes while CaCl_2_ failed to induce the expression of these genes (Fig. 2b).

### Calcium regulates secretion of T3SS effectors into extracellular milieu

**Fig. 3:**
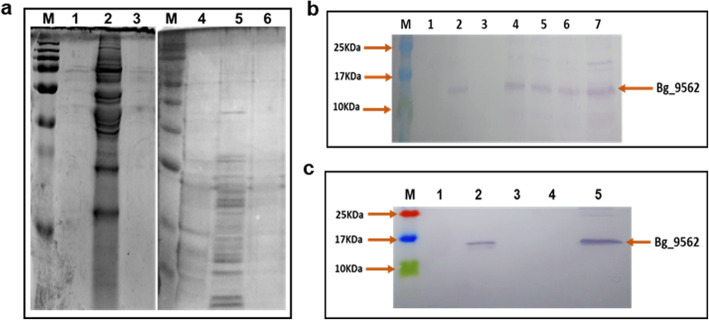
Calcium limiting condition enhances the secretion of Bg_9562 protein into extracellular milieu in a T3SS dependent manner. **a** Silver stained SDS-PAGE profile of proteins isolated from the culture supernatant of NGJ1 grown in PDB (lane 1), PDB + EGTA (lane 2) and PDB + CaCl_2_ (lane 3). While the lane 4, 5 and 6, represents the protein isolated from culture supernatants of T3SS mutant strain of NGJ1 (NGJ12) grown in PDB, PDB + EGTA and PDB + CaCl_2_, respectively. **b** Western blot analysis (using Bg_9562 specific polyclonal antibody) of proteins isolated from the culture supernatant of NGJ1 grown in PDB (lane 1), PDB + EGTA (lane 2), and PDB + CaCl_2_ (Lane 3). Lane 4, 5 and 6 represent the protein isolated from the bacterial pellet grown in PDB, PDB + EGTA and PDB + CaCl_2_, respectively. Lane 7 contains purified Bg_9562 protein obtained from the recombinant *E. coli* strain. **c** Western blot analysis of total protein isolated from the culture supernatant of wild type NGJ1 grown in absence (lane 1) or presence of EGTA (lane 2). While lane 3 and 4 represent proteins from the culture supernatant of NGJ12 grown in absence or presence of EGTA, respectively. Lane 5 contains purified Bg_9562 protein from the recombinant *E. coli* strain. M reflects pre-stained protein ladder

The phylogenetic analysis reflected that T3SS of NGJ1 is closely related to that of many plant and animal pathogenic bacteria (Additional file: Fig. S5). Under low calcium condition, most of these bacteria secrete T3SS effectors into extracellular milieu [19, 22, 25, 39, 43, 44]. We observed that NGJ1 secretes a large number of proteins into the extracellular milieu when grown under low calcium condition (EGTA supplemented PDB) while the T3SS mutant of NGJ1 (NGJ12) secreted only a limited number of proteins (Fig. 3a). On the other hand, a limited number of proteins were secreted by both NGJ1 or NGJ12 when grown in CaCl_2_ supplemented PDB (Fig. 3a). This suggested that low calcium environment triggers NGJ1 to secrete several proteins into extracellular milieu in a T3SS dependent manner.

We further tested the secretion of Bg_9562; a known T3SS effector protein of NGJ1 [14] under high (CaCl_2_ supplementation) and low calcium (EGTA supplementation) conditions. Western blot analysis using Bg_9562 specific antibody revealed the protein to be present in the bacterial pellet as well as cell-free culture supernatant, when NGJ1 was grown in low calcium condition (Fig. 3b). However, the Bg_9562 protein was not detected in the supernatant under high calcium environment (Fig. 3b). Moreover, the T3SS mutant of NGJ1 (NGJ12) was unable to secrete Bg_9562 protein under both low and high calcium conditions (Fig. 3c).

**Fig. 4:**
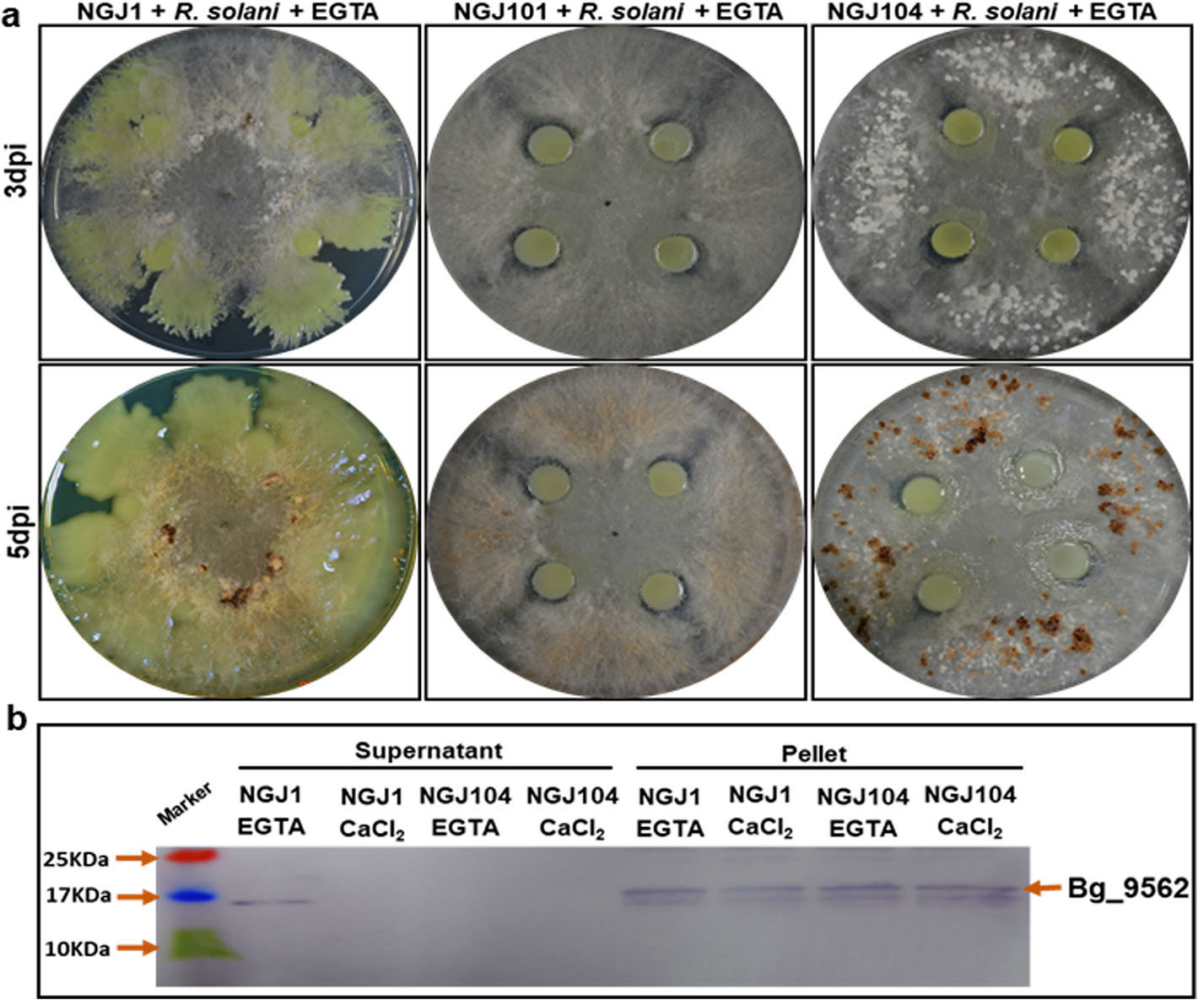
Mycophagous behaviour and secretion profile of T3SS signal deleted variant of Bg_9562 in NGJ1 strains. **a** Mycophagous activity of NGJ1 and its variant strains on *R. solani* on PDA plates. The Bg_9562 mutant (NGJ101) and T3SS signal deleted variant Bg_9562 expressing strain (NGJ104) were mycophagy deficient while the wild type NGJ1 showed enhanced mycophagy in presence of EGTA. **b** Western blot analysis (using Bg_9562 specific polyclonal antibody) of proteins isolated from the culture supernatant and bacterial pellets of NGJ1 and NGJ104 grown in PDB broth with EGTA/CaCl_2_ supplementation. Similar results were obtained in at least three independent biological experiments and only representative photographs are shown

Bg_9562 protein harbors a potential T3SS secretion signal (9 amino acids at N-terminus) which is conserved in different *B. gladioli* strains but not in other closely related bacteria (Additional file: Fig. S6). As reported previously, the Bg_9562 mutant NGJ1 (NGJ101) was defective in mycophagy [14]. Complementation of NGJ101 with full length copy of Bg_9562 gene fully restored mycophagous ability in NGJ102 while complementation with signal deleted variant of Bg_9562 failed to restore mycophagy in NGJ104 (Additional file: Fig. S7). Notably even under low calcium condition, NGJ104 failed to exhibit mycophagy (Fig. 4a). Western blot analysis revealed that although NGJ104 produces the variant Bg_9562 protein but was unable to secrete it into extracellular milieu, even in the presence of EGTA (Fig. 4b).

### Identification of calcium regulated candidate T3SS effectors of NGJ1

Using LC-MS/MS proteome analysis, we compared the EGTA induced secretome of NGJ1 and NGJ12. In this process, we identified 102 proteins to be secreted by NGJ1 but not by NGJ12 in the presence of EGTA. Computational analysis using EffectiveT3 [an online T3SS prediction tool, [45], predicted many of them (*n* = 80) to have a potential T3SS secretion signal (Additional file: Table S5). Notably, most of these proteins (*n* = 75) were not secreted by NGJ1 when grown in the presence of CaCl_2_ and hence we considered them as bonafide T3SS effectors of NGJ1 (Additional file: Table S5). The presence of Bg_9562 (a known T3SS effector of NGJ1) and orthologs of some previously known T3SS effectors of other bacteria were noteworthy amongst them. We selected a few of them with potential hydrolytic (endo β-1, 3-glucanase, peptidase S49, ribonuclease, etc.) or signaling (sensor his-kinase) functions and studied their gene expression during 24 h and 48 h of NGJ1 growth in presence or absence of *R. solani* mycelia in PDB. The RT-PCR analysis revealed them to be significantly upregulated during 48 h of confrontation with *R. solani* (Additional file: Fig. S8). Previous studies have suggested that some of the bacterial glucanases have antifungal activity [46–49]. Considering that an endo β-1, 3-glucanase gene of NGJ1 is upregulated during mycophagous interaction with *R. solani*, we endeavored to characterize its role during mycophagy.

### An endo-β-1, 3-glucanase, a potential T3SS effector of NGJ1 is required for mycophagous ability

**Fig. 5:**
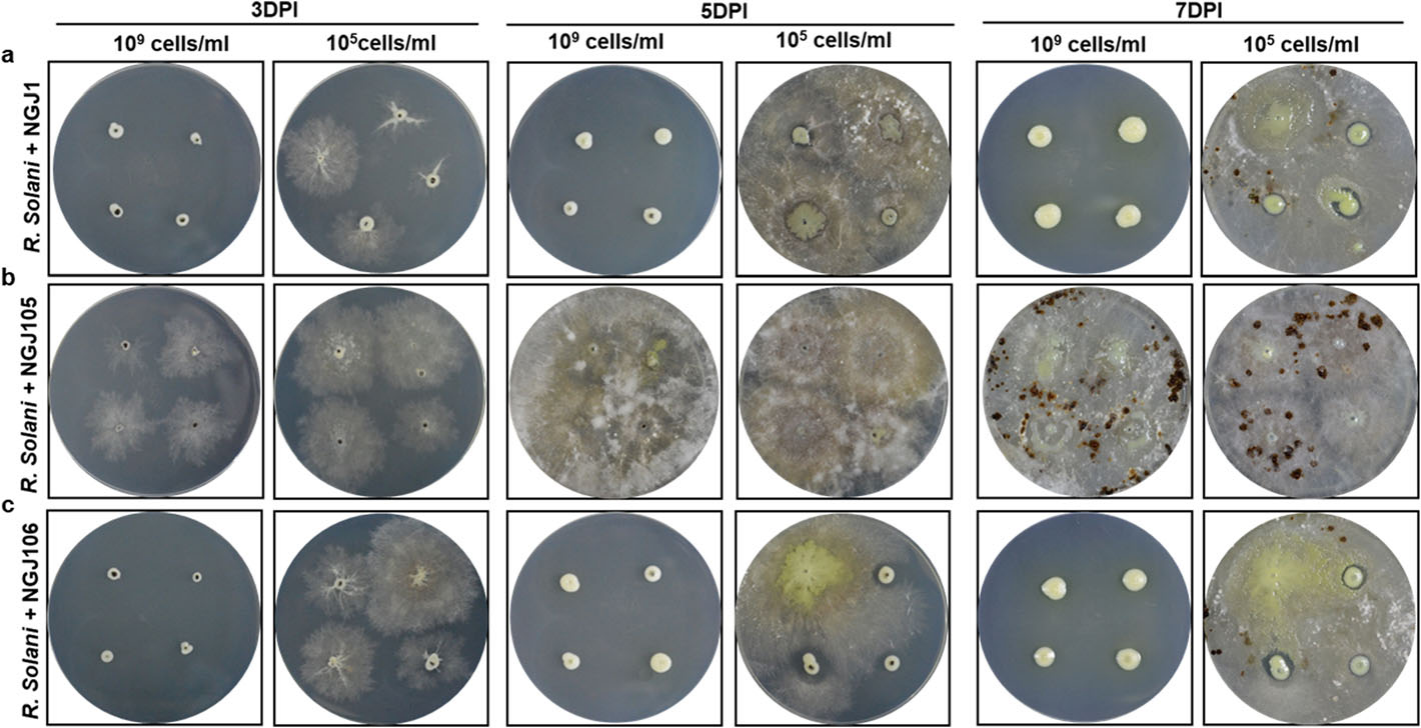
An endo-β-1, 3- glucanase is required for efficient mycophagous ability of NGJ1. The mycophagous behavior of (**a**) NGJ1, (**b**) NGJ105, the endo-β-1, 3- glucanase mutant strain and (**c**) NGJ106, the complementing strain on *R. solani* on PDA plates, at different time points. The treatment with 10^9^ cells/ml concentration of NGJ1 or NGJ106 completely prevented sclerotial germination. However, the sclerotia treated with 10^5^ cells/ml of NGJ1 or NGJ106 initially germinated but subsequently the bacteria grew over the fungal biomass. Notably, at both 10^9^ cells/ml as well as 10^5^ cells/ml concentration, the endo-β-1, 3- glucanase mutant strain (NGJ105) failed to prevent the *R. solani* sclerotia. Moreover, at high concentration (10^9^ cells/ml), limited NGJ105 growth was observed over fungal biomass. Similar results were obtained in at least three independent experiments

The phylogenetic analysis revealed that the endo-β-1, 3- glucanase of NGJ1 has similarity with that of various *Burkholderia* sp. as well as some of the previously reported glucanases [50] of other bacteria (Additional file: Fig. S9). We disrupted the endo-β-1, 3- glucanase gene of NGJ1 using homologous recombination and analyzed the mutant strain (NGJ105) for its mycophagous ability on *R. solani*. As reported earlier [14], treatment with higher concentration (10^9^ cells/ml) of NGJ1 completely inhibited the germination of *R. solani* sclerotia. While, upon treatment with lower dilution (10^5^ cells/ml) of NGJ1, the sclerotia initially germinated but subsequently the bacteria foraged over fungal biomass (Fig. 5a). On the other hand, the NGJ105 failed to inhibit the germination of *R. solani* sclerotia at both the dilutions (10^9^ or 10^5^ cells/ml) (Fig. 5b). Also, number of secondary sclerotia produced upon NGJ105 confrontation was significantly higher compared to that observed upon confrontation with NGJ1 (Additional file: Table S6). Notably, upon longer incubation (7 dpi onwards) we observed NGJ105 (especially when treated at 10^9^ cells/ml) to have slowly started foraging over fungal mycelia. However, the extent of mycophagy by NGJ105 remained significantly less compared to that of NGJ1 (Fig. 5b). Further, we complemented NGJ105 with ectopic expression of the β*-1, 3- glucanase* gene using a broad host range plasmid (pHM1) and observed the complemented strain (NGJ106) to demonstrate mycophagy on *R. solani*, similar to that of NGJ1 (Fig. 5c).

## Discussion

Bacterial type III secretion system (T3SS) is known to play an important role during bacterial interaction with plants and animals [16, 51]. However, knowledge about its involvement in bacterial-fungal interactions is limited. In a previous study, we have demonstrated that a rice associated bacterium *Burkholderia gladioli* strain NGJ1 utilizes T3SS to feed on fungi [14]. In the present study, we observed that T3SS apparatus as well as effector encoding genes of NGJ1 are upregulated during mycophagous interaction with *R. solani*. Notably, the T3SS of many gram-negative bacteria has been found to be regulated in calcium dependent manner [19, 33, 43, 52]. Our analysis revealed that calcium concentration is significantly reduced in the NGJ1-*R. solani* confrontation media. This suggested that a low calcium environment is created during interaction of NGJ1 with *R. solani,* which in turn favors mycophagy. In support of this, we observed that calcium limiting condition created by EGTA supplementation promotes mycophagy in a T3SS dependent manner. On the other hand, presence of CaCl_2_ (imparting high calcium condition) prevents NGJ1 to exhibit mycophagy. It is worth mentioning that EGTA has been extensively used as an efficient calcium ion chelator to create a low calcium environment [19, 25, 27, 28]. The possibility that EGTA may chelate other cations and thereby influence bacterial mycophagy cannot be ruled out. However, our data suggest that supplementation of MgCl_2_/FeCl_2_ or other cationic chelators (EDTA; a non-specific cationic chelator and dipyridyl, an iron chelator) have no effect on mycophagy. Also, it is worth mentioning that presence of these divalent cationic salts and their chelators have no apparent impact on the growth of NGJ1 as well as *R. solani,* under the concentration used in this study. Taken together, we speculate that low calcium environment created by EGTA promotes mycophagy while high calcium environment inhibits it. As we observed calcium dependent regulation of mycophagy in both PDA/PDB (chemically undefined media) and CDA/CDB (defined media), there is only marginal possibility that additional factors present in confrontation media may be influencing mycophagy.

EGTA mediated low calcium condition is known to trigger secretion of T3SS effector proteins into extracellular milieu in different bacterial systems [19, 24, 25, 32, 53, 54]. For instance, EGTA promoted secretion of T3SS effectors such as Yops (Yersinia outer proteins) in *Yersinia pseudotuberculosis*/ *Y. enterocolitica* and ExoS (Exoenzyme S) in *Pseudomonas aeruginosa* without host cell contact into the extracellular milieu [25, 43, 54–56]. In consistence with these studies, we observed that low calcium environment triggers NGJ1 to secrete a large number of proteins into extracellular milieu, in a T3SS dependent manner. As many of these secreted proteins (*n* = 75) contain potential T3SS secretion signal and their secretion gets prevented under high calcium environment, we considered them as bonafide T3SS effectors. The presence of a previously known T3SS effector of NGJ1 i.e. Bg_9562 (prophage tail-like protein that is required for mycophagy) [14], as well as orthologs of some known T3SS effectors in other bacterial systems (Cytidyl kinase, Serine threonine kinase, Cysteine desulfurase, alkyl hydroperoxide reductase) [32, 57–61] were noteworthy in the list.

Western blot analysis reinforced that calcium limiting condition promotes secretion of Bg_9562 in a T3SS dependent manner. We observed the presence of a T3SS secretion signal at N-terminus of Bg_9562 and found it to be important for calcium dependent secretion of the protein as well as mycophagous ability of NGJ1. Interestingly, the T3SS signal sequence is conserved in different Bg_9562 orthologs in other *B. gladioli* strains but not in other related *Burkholderia* and *Paraburkholderia sp*. Considering the above, we anticipate that *B. gladioli* strains have evolved Bg_9562 orthologs as T3SS effector. Previously, we had reported that although Bg_9562 does not contain any lytic or toxic domains, but treatment with the purified protein causes severe deformation in *R. solani* mycelia [14]. Leakage of cytosolic components and shrinkage of cell membranes were apparent in protein treated mycelia. Considering these observations, we anticipate that presence of Bg_9562 into the extracellular milieu may facilitate fungal cells lysis. Moreover, this may cause leakage of fungal metabolites into the extracellular environment which may be utilized by NGJ1 bacterium as a nutrient source during mycophagous interaction. In this context, it is worth mentioning that mycophagous bacteria are capable of utilizing fungal biomass as a source of nutrition [9, 62].

As under low calcium condition various hydrolyzing enzymes (glucanase, phosphatases, and ribonucleases), porins and phage related proteins etc. of NGJ1 get secreted into the extracellular milieu, we anticipate that they may contribute in fungal cell lysis. Here it is worth mentioning that bacterial glucanase like proteins are reported to have potent antifungal activity [46, 49, 63]. We observed that a potential T3SS effector endo β*-1, 3- glucanase* is upregulated during mycophagy. Disruption of the gene partially compromised the ability of mutant bacterium (NGJ105) to forage over *R. solani* while the ability gets restored upon complementation with the ectopic expression of the endo β*-1, 3- glucanase* gene*.* During initial days of interaction, the NGJ105 mutant was completely deficient in mycophagous ability, however at later stages it slowly started foraging over fungi. Such delayed mycophagous response of NGJ105 suggests that mycophagy is a multifactorial phenomenon and presence of other hydrolytic enzymes/Bg_9562 protein might enable the bacterium to establish mycophagy during longer incubation. It is also possible that additional factors like abundance of fungal biomass and exhaustion of nutrients in the confrontation media may be associated with delayed mycophagous response in the mutant bacterium.

## Conclusion

**Fig. 6:**
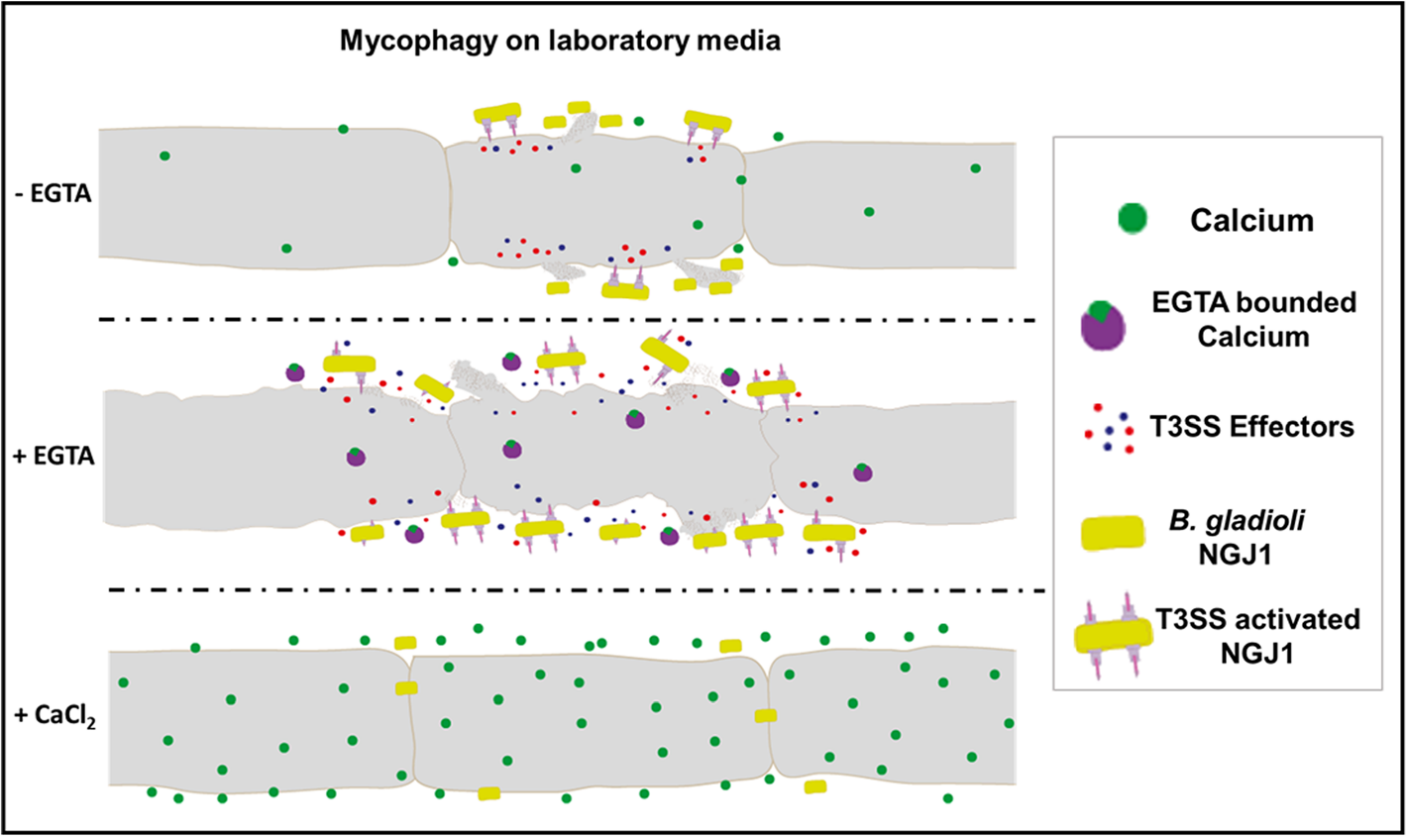
Proposed model of calcium regulated mycophagous activity of *B. gladioli* strain NGJ1. During mycophagous interaction with *R. solani*, the T3SS of NGJ1 gets activated This facilitates the delivery of various effector molecules including Bg_9562 into fungal cells in a T3SS dependent manner, which in turn is required for mycophagy. The low calcium environment created by EGTA, deregulates the T3SS and triggers the secretion of various T3SS effectors of NGJ1 into the fungal cells as well as extracellular milieu. The presence of a cocktail of potent cell wall lytic enzymes/proteins as well as Bg_9562 protein into the extracellular milieu may facilitate efficient fungal cell lysis and leakage of fungal metabolites. NGJ1 may utilize these fungal metabolites as a nutrient source to promote its growth during mycophagy. On the other hand, presence of CaCl_2_ prevents the induction of T3SS even in presence of fungi (during mycophagous interaction of NGJ1 with *R. solani*). Under this scenario, the T3SS effectors are neither delivered into the fungal cells nor secreted into the extracellular milieu. The bacterium is unable to lyse fungal membrane/release fungal metabolites and is compromised in foraging over fungal biomass

Overall our study suggests that confrontation of NGJ1 with *R. solani* creates a low calcium environment. This potentially activates T3SS apparatus assembly in NGJ1 and facilitates the delivery of effector molecules into the fungal cells to establish mycophagy. However, under calcium limiting condition T3SS apparatus of NGJ1 gets deregulated and secretion of various T3SS effectors into extracellular milieu is triggered. The presence of potent hydrolytic enzymes/proteins into extracellular milieu may facilitate efficient fungal cell lysis and release of fungal metabolites that potentially serve as nutrient source for NGJ1 during mycophagy (Fig. 6).

## Material and methods

### Growth conditions

The bacterium *Burkholderia gladioli* strain NGJ1 and its derivative strains were grown on PDA (Potato Dextrose Agar; Himedia, India) or CDA (Czapek dox agar; Sucrose: 3%, NaNO3: 0.2%, K2PO4: 0.1%, MgSO4: 0.05%, KCl: 0.05%, FeSO4: 0.001% and Agar: 1.5%; Himedia, India) plates at 28 °C. Whenever required, the media was supplemented with different antibiotics (Kanamycin: 50 μg/ml; Rifampicin: 50 μg/ml and Spectinomycin: 50 μg/ml). The fungus *Rhizoctonia solani* strain BRS1 was grown on PDA or CDA plates at 28 °C. Generally *R. solani* produces plenty of sclerotia, the spore-like resting structures on PDA/CDA plates. Germination of sclerotia produces mycelia which form secondary sclerotia at maturity. The bacterial strains and plasmids used in this study are listed in Additional file: Table S7.

### Confrontation assays of NGJ1 with *R. solani* in presence of different cationic salts

The bacterial-fungal confrontation assay was performed as described in [64]. Briefly, *R. solani* sclerotia were placed at the center of PDA or CDA plates supplemented with different concentrations (1 mM, 4 mM, and 10 mM) of various divalent cationic salts (MgCl_2_, CaCl_2_, FeCl_2_) or their chelators; EDTA (a divalent cation chelator), EGTA (a high-affinity calcium chelator) and Dipyridyl (an iron chelator). Further, 24 h pre-grown culture (20 μl of 10^9^ cells/ml) of NGJ1 was spotted at four corners, equidistant from the center on each plate. The plates were incubated at 28 °C and routinely monitored for mycophagy. The abundance of NGJ1 on fungal biomass was estimated by serial dilution plating on PDA followed by colony counting, at different time intervals. Further, the number of secondary sclerotia of *R. solani* produced on confrontation plates, with or without EGTA/CaCl_2_ supplementation was counted. Also, viability of the secondary sclerotia collected from confrontation plates was tested by growing them on fresh PDA plates [64].

For confrontation in liquid media, fungal sclerotia were initially grown for 48 h in 10 ml of PDB/CDB (Himedia, India) broth at 28 °C with constant shaking (200 rpm) to obtain mycelial mass. Subsequently, 1% of 10^9^ cells/ml NGJ1 culture was inoculated into the pre-grown fungal mycelia and different concentration of CaCl_2_ (10 mM, and 20 mM) was added into the media. After various time intervals (24 h and 48 h) of co-cultivation at 28 °C, bacterial growth was estimated by dilution plating on PDA plates, followed by colony counting. As a control, the bacterial growth in PDB/CDB media in the absence of *R. solani* mycelia was estimated.

### Sclerotial growth inhibition assay

*R. solani* sclerotial growth inhibition assay was performed as described earlier [64]. Briefly, 10^9^ cells/ml, as well as 10^5^ cells/ml concentration of NGJ1/its derivative strains, were used to treat freshly collected *R. solani* sclerotia from PDA plates. After 24 h of treatment, the sclerotia were washed with sterile Milli-Q water and transferred onto fresh PDA plates. Upon incubation at 28 °C, the mycelial growth was monitored.

### ICP-MS analysis

The 48 h pre-grown *R. solani* mycelia were inoculated with 1% overnight grown NGJ1 culture in 10 ml PDB and incubated at 28 °C. Upon 24 h and 48 h of co-cultivation, the supernatant was collected by centrifugation. Subsequently, upon filtration through 0.2 μm filter (AXIVA syringe filter), the cell-free culture supernatant was subjected to ICP-MS (Inductively Coupled Plasma Mass Spectrometry) analysis at the central research facility (CRF) of IIT-Delhi (crf.iitd.ac.in/facility-icp-ms) and metabolomics facility of NIPGR, New Delhi; following their standardized protocol to quantify calcium ions.

### Bacterial gene expression analysis

The *R. solani* mycelia were removed from confrontation media by filtering them using Mira cloth. The bacterial cells (NGJ1 or its variants) were pelleted down and total RNA was extracted from the pellet using Qiagen RNA Extraction Kit as per manufacturer’s instructions. 1 μg of total RNA was used for cDNA synthesis using Verso cDNA Synthesis Kit (Thermo Fisher Scientific Inc.). The nucleotide sequences of various NGJ1 genes were downloaded from Burkholderia Genome Database [65] and primers were designed using PRIMER 3 software (http://frodo.wi.mit.edu/primer3/)**.** The list of primers used in this study is represented in Additional file: Table S8. The real-time expression analysis was carried out using Brilliant III Ultra-Fast SYBR Green Master Mix (Agilent Technologies) on ABI 7900HT™ Real-time PCR (Applied Biosystems). 16S rRNA gene sequence of NGJ1 was used as a reference gene to normalize gene expression and the relative expression of each gene with respect to control was calculated using ΔΔCt method [66].

### Construction of T3SS signal deleted variants of Bg_9562

The T3SS signal sequence in Bg_9562 protein was predicted by using an online (www.modlab.org) tool [67]. The analysis reflected that first 9 amino acids sequence present at the N-terminal region of the protein is a potential T3SS signal. The conservation of the T3SS signal was studied in various orthologs of Bg_9562 protein in different *B. gladioli* strains as well as other closely related bacteria by multiple sequence alignment tool; Clustal Omega [68]. Primers were designed to PCR amplify the Bg_9562 gene fragment (27 bp to 333 bp), so that the first 27 nucleotides (reflecting 9 aa at N-terminal region) gets deleted (Additional file: Table S8). The PCR amplified product (306 bp) was cloned into a pHM1 vector to obtain pGD5 (Additional file: Fig. S7). Further, the pGD5 was mobilized into NGJ101 (a *Bg_9562* gene mutant strain of NGJ1) by electroporation (Gene pulsar Xcell^Tm^; BioRad) as per the method described earlier [14]. The transformed colonies were selected on Kanamycin and Rifampicin supplemented KBA (King’s medium B Base; Himedia, India) plates. Upon PCR verification using gene-specific and M13 vector-specific primers listed in Table S8, the complimenting strain with the T3SS signal deleted Bg_9562 gene variant was named as NGJ104 and used for further studies.

### Protein isolation

The culture supernatant of 100 ml culture of wild type, as well as T3SS mutant strain of NGJ1 grown for 24 h in PDB with EGTA (4 mM)/CaCl2 (10 mM) supplementation or without any supplementation was collected. The proteins were precipitated using TCA (trichloroacetic acid; 12%) and washed twice with 70% ethanol, before being re-suspended in PBS buffer (10 mM PBS; pH -7.4). The isolated proteins were resolved on SDS-PAGE and visualized by silver staining. The crude protein extract was also isolated from the bacterial pellet by grinding them using liquid N_2_ and dissolving in 30 ml of buffer (10 mM PBS; pH -7.4, 1 mM lysozyme, and 1 mM PMSF) followed by centrifugation to remove cell debris.

### Western blot analysis

The bacterial proteins were resolved on SDS-PAGE gel (15%) followed by electro-blotting onto polyvinylidene fluoride (PVDF) membrane and western blot analysis was performed as described in [14]. The anti-Bg_9562 (1:50000 dilution) antibody was used as a primary and alkaline phosphatase-conjugated anti-rabbit IgG (Sigma) was used as secondary antibody (1:10000 dilution). The purified Bg_9562 protein from the recombinant *E. coli* strain was used as a positive control in this analysis. Due to the presence of His-tag, the recombinant protein is apparently large than that of the expected size of native Bg_9562 protein of *B. gladioli* strain NGJ1.

### LC-MS/MS-based proteome analysis

Total protein isolated from the culture supernatant (as described above) was further precipitated by adding pre-cooled acetone (320,110, Sigma) and resuspended in 8 M urea. After incubating the protein suspension in 20 mM DTT solution at 60 °C for 1 h, the trypsin digestion was performed at 37 °C for 18 h in 100 mM tri-ethyl ammonium bicarbonate (pH 8, T7408, Sigma) solution. After ZipTip desaltation (Cat. ZTC18S960, Millipore), the digested peptides were dissolved in 12 μl of 0.1% formic acid and analyzed by nanoLC-MS/MS using an Eksigent ekspert™ nanoLC 425 system coupled to AB Sciex TripleTOF® 6600 System with the service support of Xcelris™ Labs Limited (Ahmedabad, India). Raw data files were converted to Mascot Generic Format (MGF) format using msconvert and searched against the UniProt, NCBI and common MS contaminant database using Mascot 2.5 (Matrix Science) Software. The proteins, thus identified, were subjected to T3SS effector prediction using an online tool; EffectiveT3 [45].

### Phylogenetic analysis

The amino acid sequences of HrcC (an important constituent of the T3SS apparatus) of different bacterium used in the study were downloaded from NCBI database [69]. Phylogenetic tree was constructed by Maximum Likelihood method and Tamura-Nei model using MEGAX software. Neighbor-Joining clustering and BioNJ algorithms were applied to a matrix of pairwise distances estimated using the maximum composite Likelihood (MCL) approach to construct an initial tree for the heuristic search. Similarly, phylogenetic tree reflecting the similarity of endo-β-1, 3- glucanase of NGJ1 with that of previously reported glucanases of other bacteria [50] was constructed.

### Deletion and complementation of endo-β-1, 3- glucanase gene in NGJ1

A partial fragment of the endo-β-1, 3- glucanase gene (435 bp) was PCR amplified from *B. gladioli* strain NGJ1 using gene-specific primers (Additional file: Table S8) and cloned into pK18mob vector to obtain pGD6 plasmid. The pGD6 was mobilized into NGJ1 by electroporation, as described above. The mutants were confirmed through PCR using gene-specific flanking as well as M13 vector-specific primers listed in Additional file: Table S8. The positive mutant strain, thus obtained was named as NGJ105. For complementation, the full-length CDS region of the gene (1.8 kb) was PCR amplified from NGJ1 gDNA by using full-length gene-specific primers (Additional file: Table S8) and cloned into pHM1 vector to obtain pGD7 plasmid. The pGD7 plasmid was electroporated into NGJ105 strain to obtain NGJ106. The mycophagous as well as sclerotial growth prevention ability of NGJ105, NGJ106 and NGJ1 were analyzed, following the protocol as described above.

## Supplementary information

**Additional file 1: Fig. S1.** Effects of different divalent cations on bacterial mycophagy. Confrontation of NGJ1 with *R. solani* on PDA plates either containing 10 mM concentration of different divalent cationic salts (CaCl_2_, MgCl_2_ and FeCl_2_) or 4 mM concentration of their respective chelators (EGTA, EDTA and Dipyridyl). The mycophagous ability was enhanced on EGTA containing plates while inhibited on CaCl_2_ containing plates. However, other divalent cationic salts (MgCl_2_ and FeCl_2_) as well as their chelators (EDTA and Dipyridyl) did not alter mycophagous ability. Notably, the presence of different divalent cationic salts and their respective chelators did not alter the growth of *R. solani* as well as NGJ1. Similar results were obtained in at least three independent biological experiments and only representative photographs are shown. **Fig. S2.** Mycophagous behaviour of NGJ1 on *R. solani* in CaCl_2_ supplemented semi-synthetic minimal media. (A) NGJ1 shows mycophagy on CDA (without supplementation) plates and is able to forage over the fungal mycelium. While supplementation of 5 mM, 10 mM and 20 mM concentration of CaCl_2_ onto CDA plates gradually reduced the mycophagy. (B) Bacterial abundance in CDB broth with or without CaCl_2_ supplementation. NGJ1 showed limited growth in CDB broth while the growth was enhanced in presence of *R. solani* mycelia. However, supplementation of different concentration of CaCl_2_ suppressed the bacterial growth in CDB containing *R. solani* mycelia. Similar results were obtained in at least three independent biological experiments and only representative images are shown. Graphs show mean values± standard deviation. **Fig. S3.** Low calcium condition regulates mycophagy in a functional T3SS dependent manner. Confrontation of *R. solani* with NGJ1 or NGJ12, a T3SS deficient mutant strain on PDA plates with or without EGTA/CaCl_2_ supplementation at 3 dpi. Presence of EGTA or CaCl_2_ didn’t alter the mycophagy defect of NGJ12. Similar results were obtained in at least three independent biological experiments and only representative images are shown. **Fig. S4.** Expression profile of T3SS apparatus encoding genes of NGJ1. RT- PCR analysis reflected expression of T3SS apparatus encoding genes to be upregulated in presence of *R. solani* at 48 h. However presence of 10 mM and 20 mM CaCl_2_ reduced their expression. The differential expression of these genes was estimated during NGJ1 growth in presence of *R. solani* with respect to that observed in absence of *R. solani* using 16S rRNA gene as endogenous control. The experiment was independently repeated three times with minimum three technical replicates. Asterisks * and ** indicate statistical significant difference between indicated groups at *P* < 0.05 and *P* < 0.001, respectively (estimated using one-way ANOVA). Graphs show mean values ± standard deviation. **Fig. S5.** HrcC (an important constituent of T3SS) protein sequence based phylogenetic relationship of NGJ1 with other bacteria. The phylogenetic tree was constructed using maximum likelihood method. The bootstrap values are indicated at branch node. The information about calcium mediated regulation of the T3SS of different bacteria has been mentioned and the related reference has been cited. **Fig. S6.** Conservation of T3SS signal sequence in Bg_9562 orthologs. Multiple sequence alignment of orthologs of Bg_9562 protein in different bacteria revealed that potential T3SS signal (9 amino acids sequence at N-terminus) is conserved in different *B. gladioli* strains. Predicted T3SS signal sequence is highlighted in blue-square. Rsol: *Ralstonia solanacearum*; Bpse: *B. pseudomallei*; Bsta: *B. stagnalis*; Bcen: *B. cenocepacia*; Bglu: *B. glumae*; Bubo: *B. ubonensis*; Bgla: *B. gladioli*; Pbacid: *Paraburkholderia acidipaludis*; Pbterri; *Paraburkholderia terricola*. **Fig. S7.** Complementation with T3SS signal deleted variant of *Bg_9562* failed to restore mycophagy in the *Bg_9562* mutant bacterium. (A) Strategy adopted to clone T3SS signal sequence deleted (pGD5) as well as full length *Bg_9562* (pGD3). (B) Effect of *Bg_9562* mutant (NGJ101) and mutant strains complemented with pGD3 (NGJ102) and pGD5 (NGJ104) on the germination and growth of *R. solani* sclerotia. At both high (10^9^cells/ml) and low (10^5^ cells/ml) concentration, the NGJ101 as well as NGJ104 were defective in mycophagous ability. While the wild type (NGJ1) as well as NGJ102 were proficient in mycophagy. **Fig. S8.** Expression profile of potential T3SS effector encoding genes of NGJ1 during mycophagous interaction with *R. solani*. RT-PCR analysis revealed the T3SS effector encoding genes of NGJ1 to be induced during 48 h of mycophagous interaction in PDB. The differential gene expression was estimated during mycophagous interaction of NGJ1 with *R. solani* with respect to that observed during NGJ1 growth in absence of *R. solani* using 16S rRNA gene as endogenous control. The experiment was independently repeated three times with minimum three technical replicates. Asterisks * indicate significantly different at *P* < 0.001 (estimated using one-way ANOVA). Graphs show mean values± standard deviation. **Fig. S9.** Phylogenetic analysis of glucanase like proteins in *Burkholderia* sp. and various other bacteria. The amino acid sequence of proteins of different bacterial species has been obtained from NCBI and used for construction of phylogenetic tree using maximum likelihood method. The red box depicts the endo-β-1, 3-glucanase of NGJ1. The bootstrap values are indicated at branch node. **Table S1.** Mycophagous behaviour of NGJ1 on *R. solani* in presence of different cationic salts and their chelators. **Table S2.** Sclerotial formation on *B. gladioli* strain NGJ1 and *R. solani* confrontation plates upon various cationic salts supplementation. **Table S3.** Germination rate of secondary sclerotia of *R. solani* isolated from NGJ1 confrontation plates. **Table S4.** Calcium concentration measurement through ICP-MS. **Table S5.** Bonafide T3SS effector proteins of NGJ1. **Table S6.** Effect of the endo-beta- 1, 3- glucanase mutant (NGJ105) and complement (NGJ106) strains of NGJ1 on secondary sclerotia production by *R. solani.***Table S7.** Bacterial strains and plasmids used in this study. **Table S8.** Primers used in this study.

## Data Availability

All data generated or analyzed during this study are included in this article and its supplementary information file.

## Abbreviations

*B. gladioli*: *Burkholderia gladioli*
*R. solani*: *Rhizoctonia solani*
T3SS: Type III secretion system
EGTA: Ethylene glycol tetra-acetic acid
EDTA: Ethylene-diamine tetra-acetic acid
CDA: Czapek dox agar
CDB: Czapek dox broth
PDA: Potato dextrose agar
PDB: Potato dextrose broth
CFU: Colony-forming unit
ICP-MS: Inductively coupled plasma mass spectrometry
LC-MS/MS: Liquid chromatography-tandem mass spectrometry
