# Supplementary material for: Calcium regulates the mycophagous ability of Burkholderia gladioli strain NGJ1 in a type III secretion system-dependent manner
Source: BMC Microbiol. 2020 Jul 20;20:216. doi: 10.1186/s12866-020-01897-2 (PMC7372643; doi:10.1186/s12866-020-01897-2)
Supplement: Supplementary file 1 — Additional file 1: Fig. S1. Effects of different divalent cations on bacterial mycophagy. Confrontation of NGJ1 with R. solani on PDA plates either containing 10 mM concentration of different divalent cationic salts (CaCl2, MgCl2 and FeCl2) or 4 mM concentration of their respective chelators (EGTA, EDTA and Dipyridyl). The mycophagous ability was enhanced on EGTA containing plates while inhibited on CaCl2 containing plates. However, other divalent cationic salts (MgCl2 and FeCl2) as well as their chelators (EDTA and Dipyridyl) did not alter mycophagous ability. Notably, the presence of different divalent cationic salts and their respective chelators did not alter the growth of R. solani as well as NGJ1. Similar results were obtained in at least three independent biological experiments and only representative photographs are shown. Fig. S2. Mycophagous behaviour of NGJ1 on R. solani in CaCl2 supplemented semi-synthetic minimal media. (A) NGJ1 shows mycophagy on CDA (without supplementation) plates and is able to forage over the fungal mycelium. While supplementation of 5 mM, 10 mM and 20 mM concentration of CaCl2 onto CDA plates gradually reduced the mycophagy. (B) Bacterial abundance in CDB broth with or without CaCl2 supplementation. NGJ1 showed limited growth in CDB broth while the growth was enhanced in presence of R. solani mycelia. However, supplementation of different concentration of CaCl2 suppressed the bacterial growth in CDB containing R. solani mycelia. Similar results were obtained in at least three independent biological experiments and only representative images are shown. Graphs show mean values± standard deviation. Fig. S3. Low calcium condition regulates mycophagy in a functional T3SS dependent manner. Confrontation of R. solani with NGJ1 or NGJ12, a T3SS deficient mutant strain on PDA plates with or without EGTA/CaCl2 supplementation at 3 dpi. Presence of EGTA or CaCl2 didn’t alter the mycophagy defect of NGJ12. Similar results were obtained in at [file 12866_2020_1897_MOESM1_ESM.docx]

**
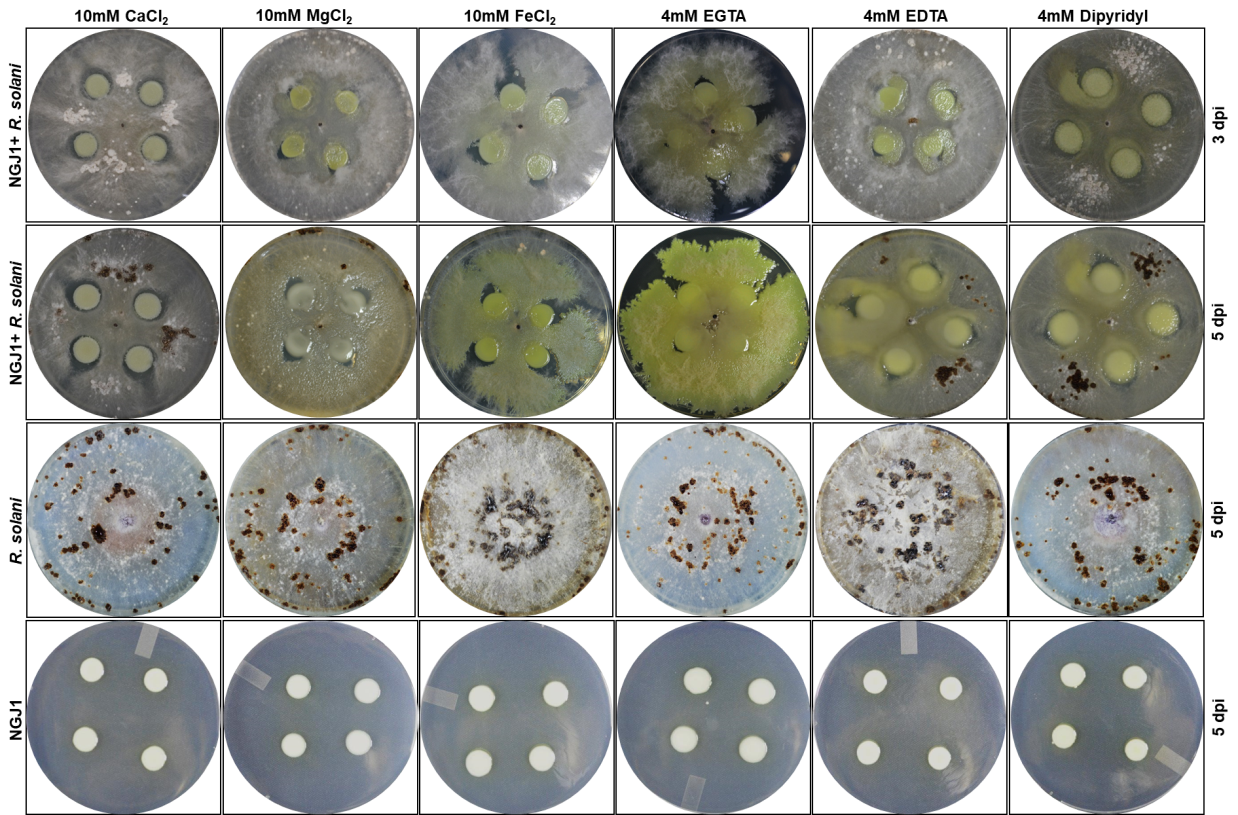
**

**FIG S1 Effects of different divalent cations on bacterial mycophagy.** Confrontation of NGJ1 with *R. solani* on PDA plates either containing 10mM concentration of different divalent cationic salts (CaCl_2_, MgCl_2_ and FeCl_2_) or 4mM concentration of their respective chelators (EGTA, EDTA and Dipyridyl). The mycophagous ability was enhanced on EGTA containing plates while inhibited on CaCl_2_ containing plates. However, other divalent cationic salts (MgCl_2_ and FeCl_2_) as well as their chelators (EDTA and Dipyridyl) did not alter mycophagous ability. Notably, the presence of different divalent cationic salts and their respective chelators did not alter the growth of *R. solani* as well as NGJ1. Similar results were obtained in at least three independent biological experiments and only representative photographs are shown.

**
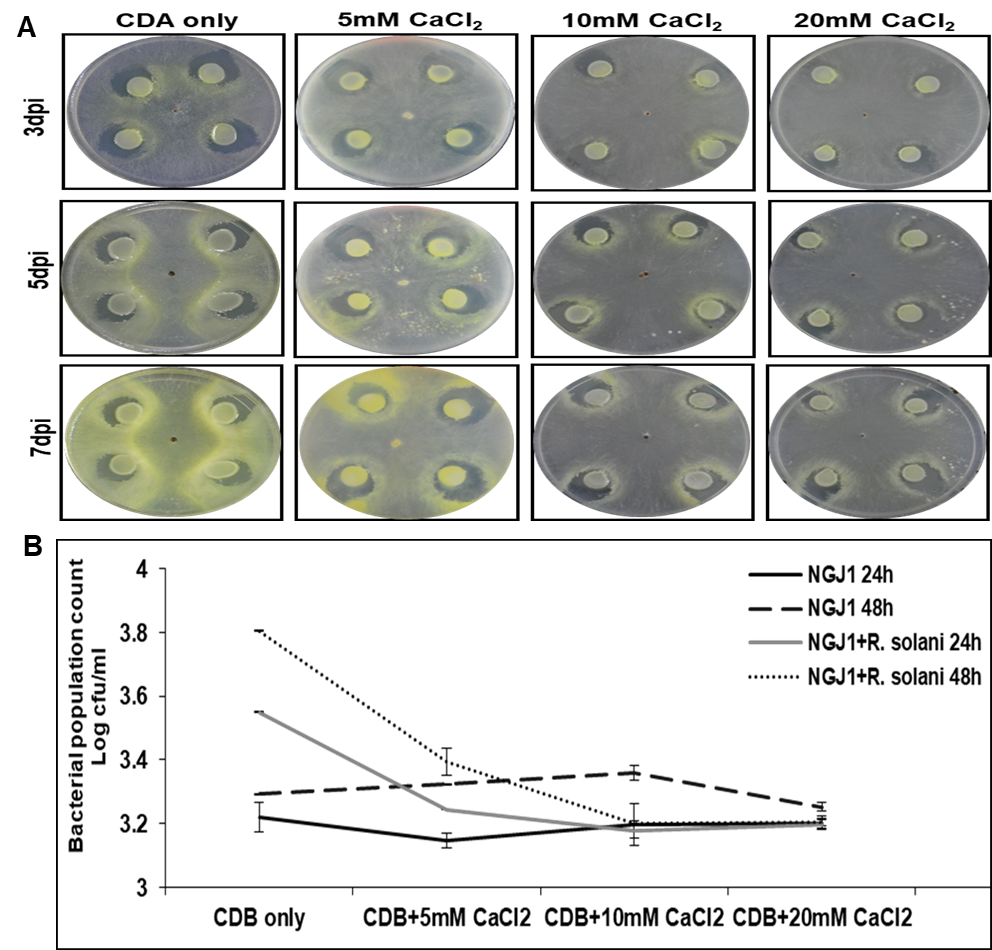
**

**FIG S2 Mycophagous behaviour of NGJ1 on *R. solani* in CaCl_2_ supplemented semi-synthetic minimal media.** (A) NGJ1 shows mycophagy on CDA (without supplementation) plates and is able to forage over the fungal mycelium. While supplementation of 5mM, 10mM and 20mM concentration of CaCl_2_ onto CDA plates gradually reduced the mycophagy. (B) Bacterial abundance in CDB broth with or without CaCl_2_ supplementation. NGJ1 showed limited growth in CDB broth while the growth was enhanced in presence of *R. solani* mycelia. However, supplementation of different concentration of CaCl_2_ suppressed the bacterial growth in CDB containing *R. solani* mycelia. Similar results were obtained in at least three independent biological experiments and only representative images are shown. Graphs show mean values± standard deviation.


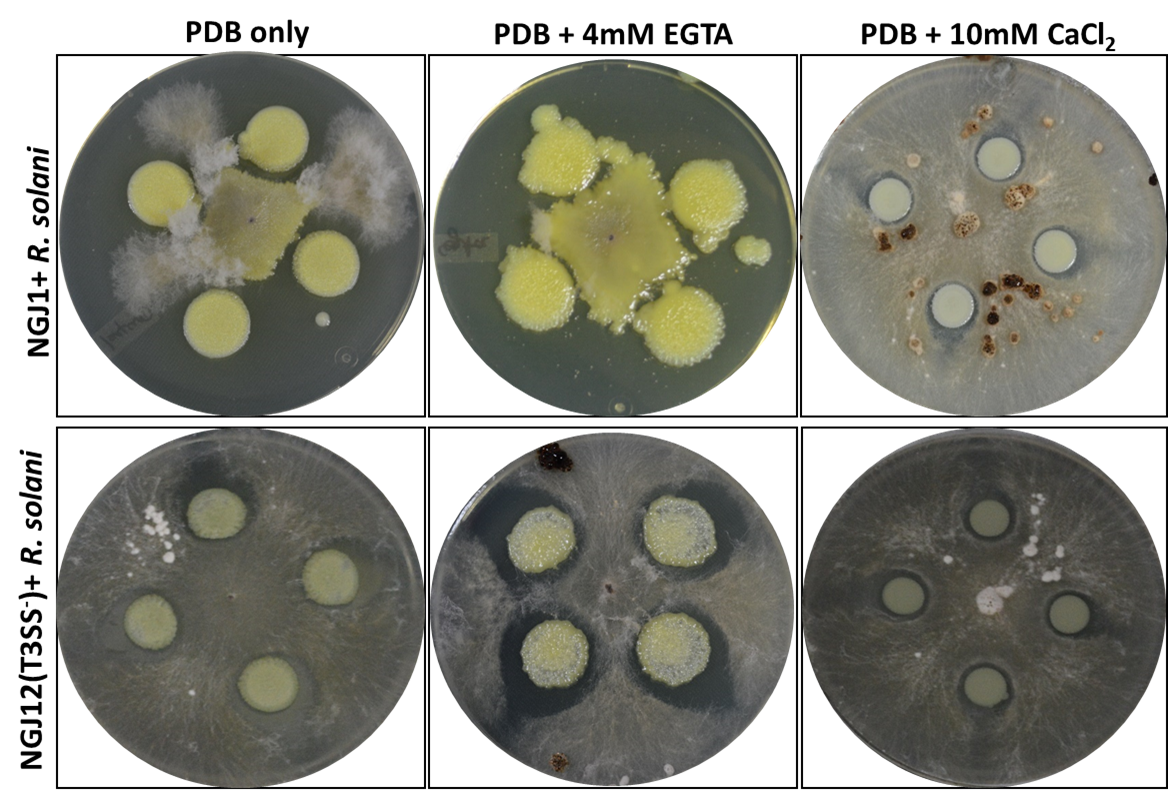


**FIG S3 Low calcium condition regulates mycophagy in a functional T3SS dependent manner**. Confrontation of *R. solani* with NGJ1 or NGJ12, a T3SS deficient mutant strain on PDA plates with or without EGTA/CaCl_2_ supplementation at 3 dpi. Presence of EGTA or CaCl_2_ didn’t alter the mycophagy defect of NGJ12. Similar results were obtained in at least three independent biological experiments and only representative images are shown.

**
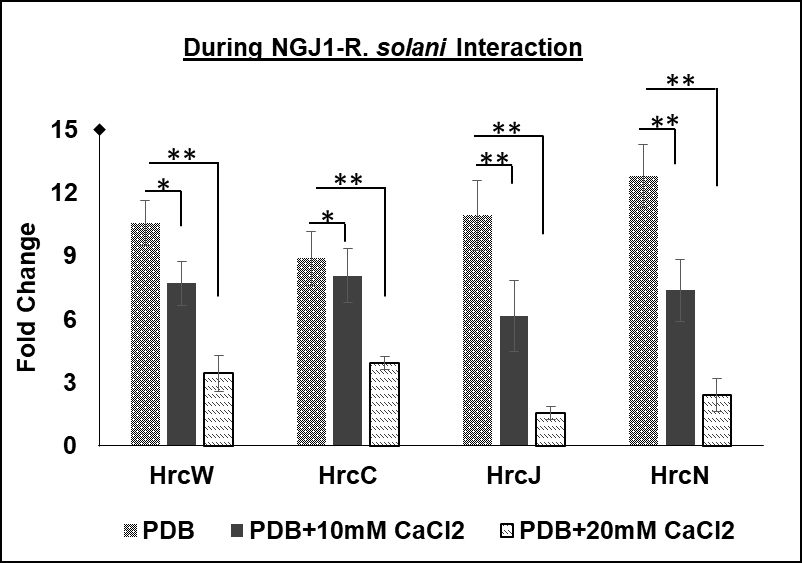
**

**FIG S4 Expression profile of T3SS apparatus encoding genes of NGJ1.** RT- PCR analysis reflected expression of T3SS apparatus encoding genes to be upregulated in presence of *R. solani* at 48h. However presence of 10mM and 20mM CaCl_2_ reduced their expression. The differential expression of these genes was estimated during NGJ1 growth in presence of *R. solani* with respect to that observed in absence of *R. solani* using 16S rRNA gene as endogenous control. The experiment was independently repeated three times with minimum three technical replicates. Asterisks * and ** indicate statistical significant difference between indicated groups at P<0.05 and P<0.001, respectively (estimated using one-way ANOVA). Graphs show mean values ± standard deviation.


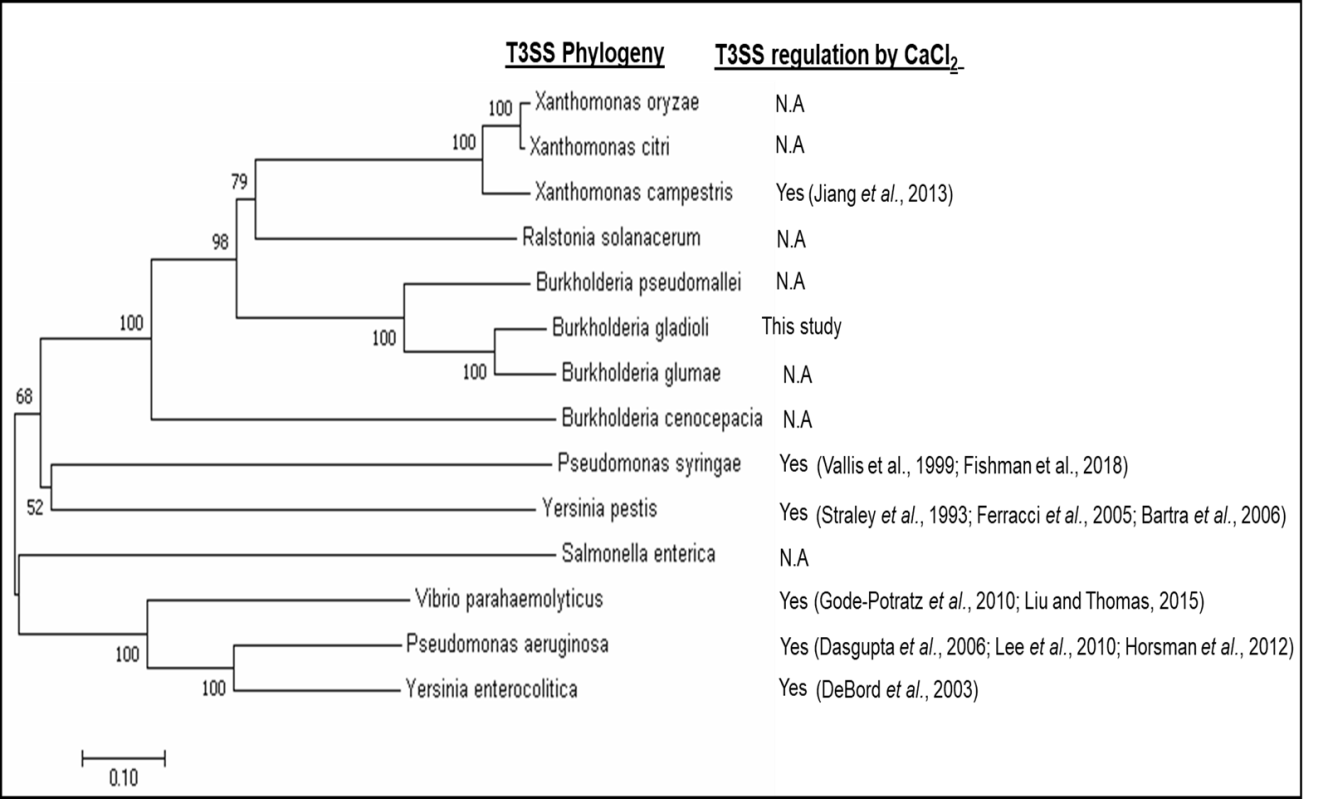


**FIG S5 HrcC (an important constituent of T3SS) protein sequence based phylogenetic relationship of NGJ1 with other bacteria.** The phylogenetic tree was constructed using maximum likelihood method. The bootstrap values are indicated at branch node. The information about calcium mediated regulation of the T3SS of different bacteria has been mentioned and the related reference has been cited.

**
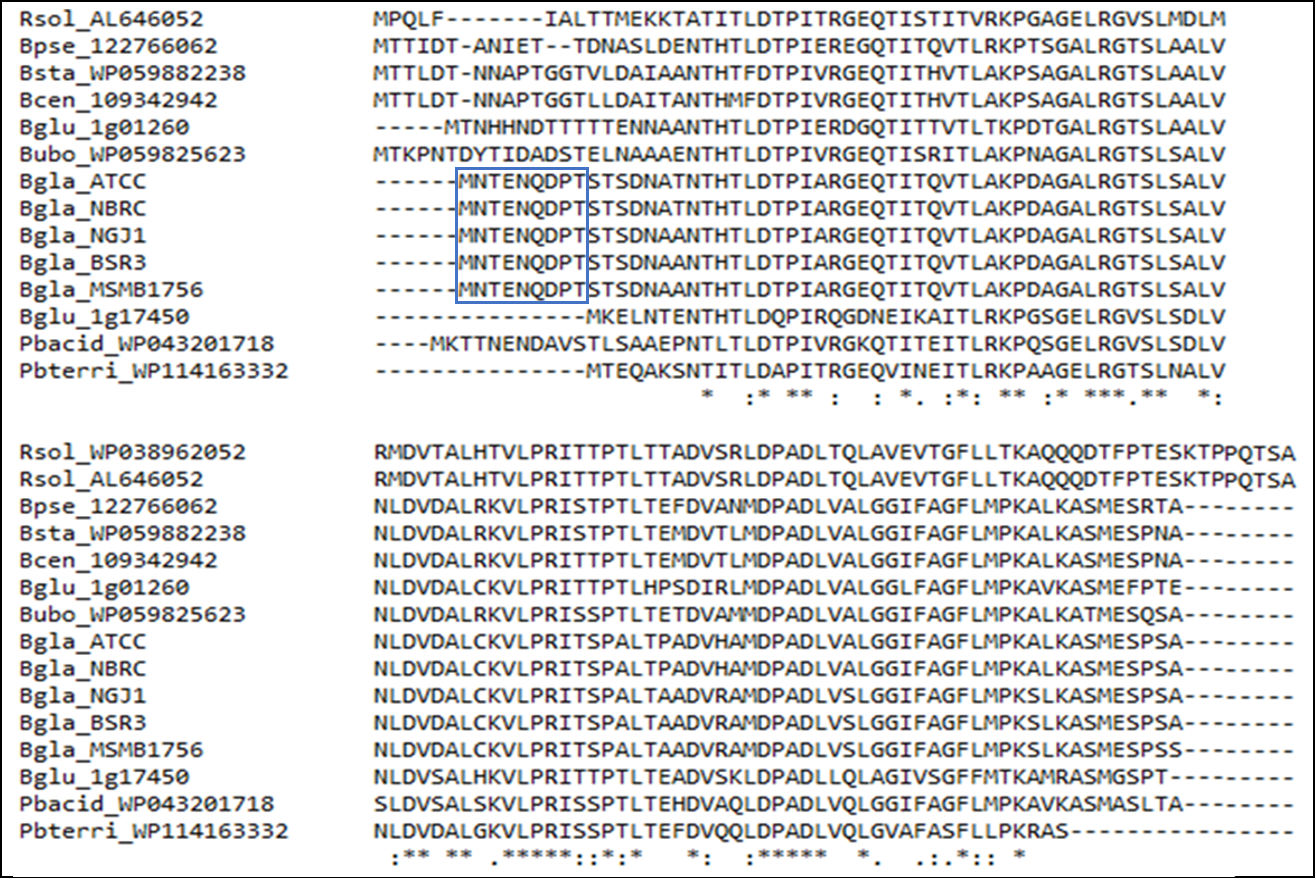
**

**FIG S6 Conservation of T3SS signal sequence in Bg_9562 orthologs.** Multiple sequence alignment of orthologs of Bg_9562 protein in different bacteria revealed that potential T3SS signal (9 amino acids sequence at N-terminus) is conserved in different *B. gladioli* strains. Predicted T3SS signal sequence is highlighted in blue-square. Rsol: *Ralstonia solanacearum*; Bpse: *B. pseudomallei*; Bsta: *B. stagnalis*; Bcen: *B. cenocepacia*; Bglu: *B. glumae*; Bubo: *B. ubonensis*; Bgla: *B. gladioli*; Pbacid: *Paraburkholderia acidipaludis*; Pbterri; *Paraburkholderia terricola*.

**
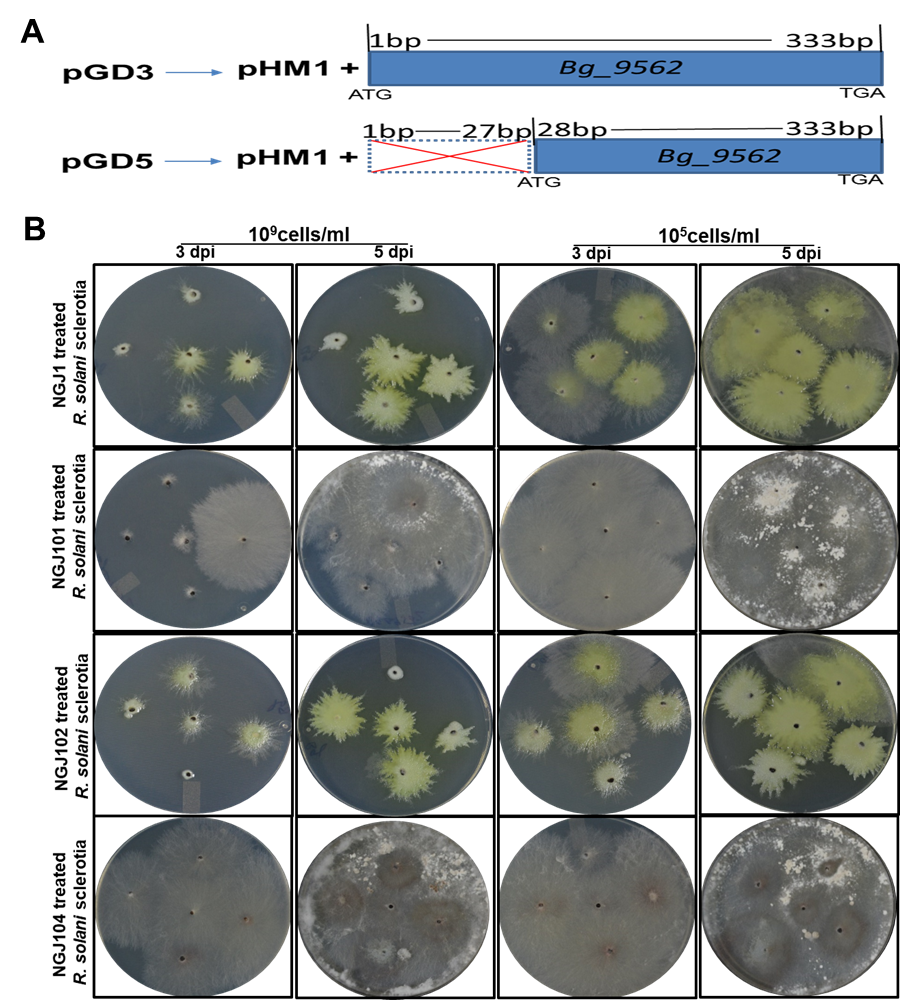
**

**FIG S7 Complementation with T3SS signal deleted variant of *Bg_9562* failed to restore mycophagy in the *Bg_9562* mutant bacterium.** (A) Strategy adopted to clone T3SS signal sequence deleted (pGD5) as well as full length *Bg_9562* (pGD3). (B) Effect of *Bg_9562* mutant (NGJ101) and mutant strains complemented with pGD3 (NGJ102) and pGD5 (NGJ104) on the germination and growth of *R. solani* sclerotia. At both high (10^9^cells/ml) and low (10^5^ cells/ml) concentration, the NGJ101 as well as NGJ104 were defective in mycophagous ability. While the wild type (NGJ1) as well as NGJ102 were proficient in mycophagy.

**
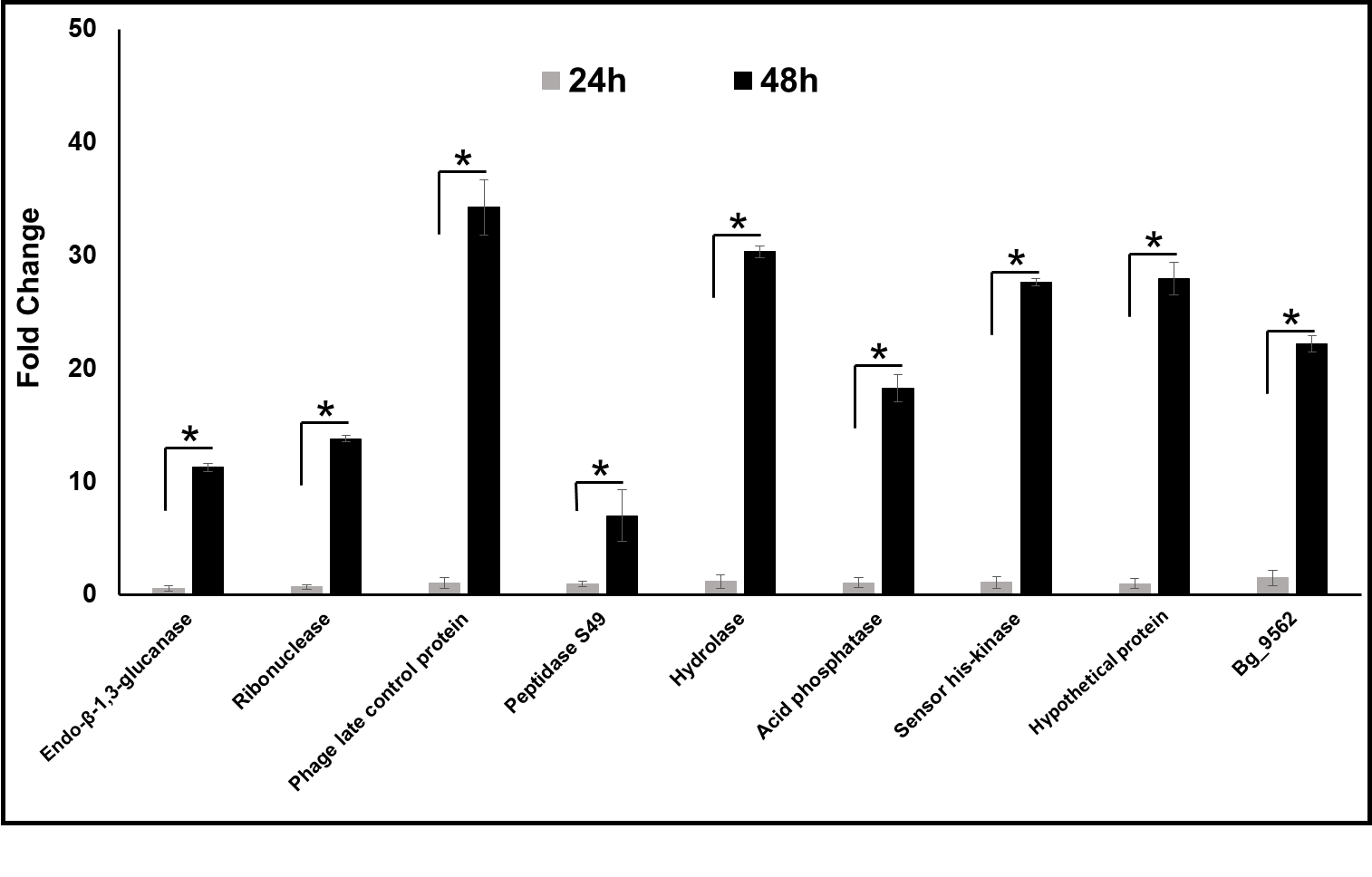
**

**FIG S8 Expression profile of potential T3SS effector encoding genes of NGJ1 during mycophagous interaction with *R. solani*.** RT-PCR analysis revealed the T3SS effector encoding genes of NGJ1 to be induced during 48h of mycophagous interaction in PDB. The differential gene expression was estimated during mycophagous interaction of NGJ1 with *R. solani* with respect to that observed during NGJ1 growth in absence of *R. solani* using 16S rRNA gene as endogenous control. The experiment was independently repeated three times with minimum three technical replicates. Asterisks * indicate significantly different at *P*<0.001 (estimated using one-way ANOVA). Graphs show mean values± standard deviation.

**
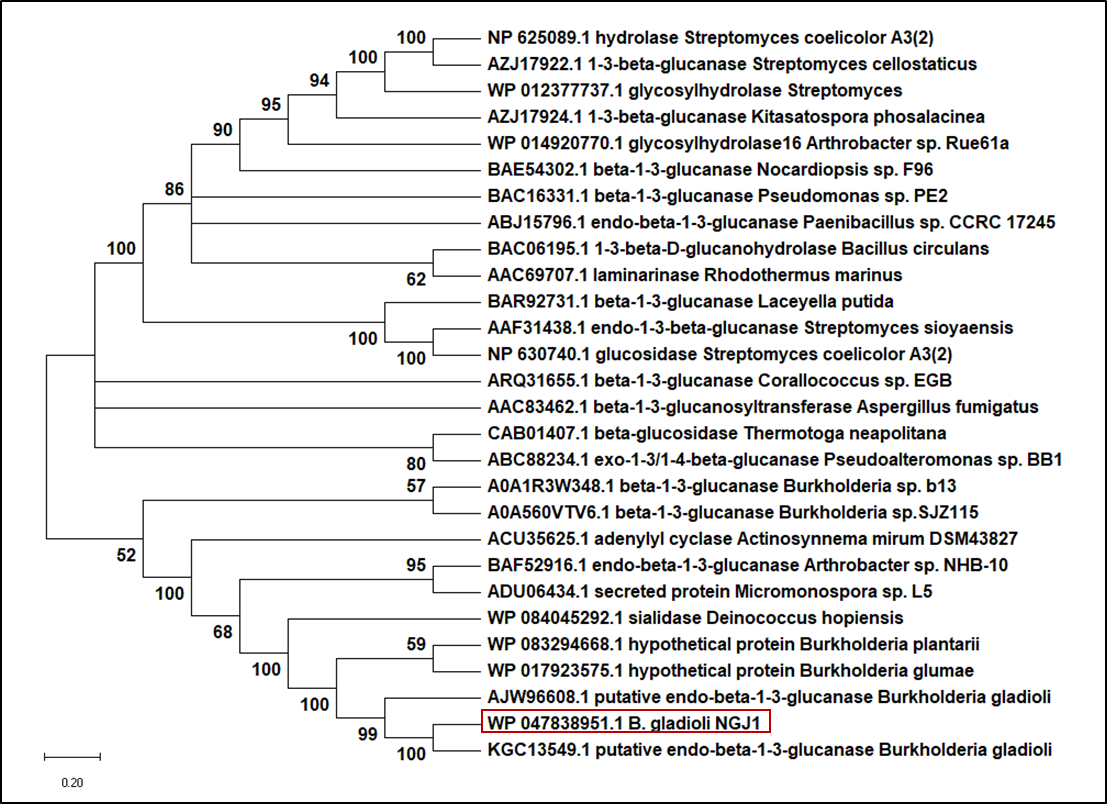
**

**Fig. S9 Phylogenetic analysis of glucanase like proteins in *Burkholderia* sp.** **and various other bacteria.** The amino acid sequence of proteins of different bacterial species has been obtained from NCBI and used for construction of phylogenetic tree using maximum likelihood method. The red box depicts the endo-β-1, 3-glucanase of NGJ1. The bootstrap values are indicated at branch node.

**Table S1. Mycophagous behaviour of NGJ1 on *R. solani* in presence of different cationic salts and their chelators**

| **Presence of cationic salts and their chelators** | **Concentration** | **Mycophagous behavior #** |
| --- | --- | --- |
| NGJ1 only | - | ++ |
| NGJ1 + CaCl_2_ | 1mM | + |
|  | 4mM | No mycophagy |
|  | 10mM | No mycophagy |
| NGJ1 + MgCl_2_ | 1mM | ++ |
|  | 4mM | ++ |
|  | 10mM | ++ |
| NGJ1 + FeCl_2_ | 1mM | ++ |
|  | 4mM | ++ |
|  | 10mM | ++ |
| NGJ1 + EGTA | 1mM | ++ |
|  | 4mM | ++++++ |
|  | 10mM***** |  |
| NGJ1 + EDTA | 1mM | ++ |
|  | 4mM | ++ |
|  | 10mM | ++ |
| NGJ1 + dipyridyl | 1mM | ++ |
|  | 4mM | ++ |
|  | 10mM | ++ |

#: symbol indicates extent of mycophagous ability of NGJ1 on *R. solani*; ‘++’ indicates level of mycophagy that is normally exhibited by NGJ1 in PDA; ‘+’ indicates comparatively less mycophagy, while ‘++++++’ indicates significantly enhanced mycophagy.

*****: At 10 mM concentration of EGTA, *R. solani* sclerotia as well as NGJ1 failed to grow, thus we could not test mycophagous interaction of NGJ1 with fungi.

**Table S2. Sclerotial formation on *B. gladioli* strain NGJ1 and *R. solani* confrontation plates upon various cationic salts supplementation.**

|  | ***R. solani* *** | | ***R. solani* + NGJ1***** | |
| --- | --- | --- | --- | --- |
|  | Sclerotia | SD **^Ŧ^** | Sclerotia | SD **^Ŧ^** |
| **PDA only** | 184.8 | 24.2 | 20.5 | 7.4 |
| **PDA + 10mM CaCl_2_** | 172.5 | 27.4 | 127.7 | 25.4 |
| **PDA + 4mM EGTA** | 132.5 | 25.4 | 2.1 | 1.2 |
| **PDA + 10mM MgCl_2_** | 148.3 | 31.9 | 24.9 | 14.7 |
| **PDA + 4mM EDTA** | 134.6 | 1.7 | 16.7 | 9.5 |
| **PDA + 10mM FeCl_2_** | 145.1 | 24.4 | 28.8 | 6.6 |
| **PDA + 4mM dipyridyl** | 128.7 | 15.2 | 34.3 | 9.0 |

***:** Average number of sclerotia from five independent replicates.

**^Ŧ:^** Standard deviation

**Table S3. Germination rate of secondary sclerotia of *R. solani* isolated from NGJ1 confrontation plates.**

|  | **Repeat-1*** | **Repeat-2*** | **Repeat-3*** |
| --- | --- | --- | --- |
| ***R. solani*+ NGJ1** | 0/5 | 0/5 | 0/5 |
| ***R. solani*+ NGJ1+ CaCl_2_** | 5/5 | 5/5 | 5/5 |
| ***R. solani* + NGJ1+ EGTA** | 0/5 | 0/5 | 0/5 |
| ***R. solani*+ NGJ1+ MgCl_2_** | 0/5 | 2/5 | 1/5 |
| ***R. solani* + NGJ1+ EDTA** | 0/5 | 1/5 | 1/5 |
| ***R. solani*+ NGJ1+ FeCl_2_** | 0/5 | 0/5 | 0/5 |
| ***R. solani* + NGJ1+ dipyridyl** | 0/5 | 1/5 | 2/5 |

*****: no of germinating/total no. of treated *R. solani* sclerotia

**Table S4. Calcium concentration measurement through ICP-MS**

| **Samples/DPI** | **24h** | | **48h** | |
| --- | --- | --- | --- | --- |
|  | Conc. [ ppb ] | Conc. RSD | Conc. [ ppb ] | Conc. RSD |
| **PDA only** | 369.6 | 1.3 | - | - |
| **PDB only** | 258.32 | 4.74 | 252.86 | 3.69 |
| **PDB +NGJ1** | 306.40 | 5.45 | 254.78 | 4.09 |
| **PDB + *R. solani*** | 286.89 | 4.33 | 262.05 | 5.02 |
| **PDB + *R. solani +*NGJ1** | 194.81 | 7.24 | 68.31 | 5.82 |

**ppb: parts per billion; RSD- Relative standard deviation**

**Table S5. Bonafide T3SS effector proteins of NGJ1**

| Locus Id | Functions | Effective T3 prediction***** | Secreted by NGJ1 in presence of EGTA (4mM)**^#^** | Secreted by T3SS mutant NGJ1 in presence of EGTA (4mM)**^#^** | Secreted by NGJ1 in presence of Calcium (10mM)**^#^** |
| --- | --- | --- | --- | --- | --- |
| WP_047836757.1 | LD-carboxypeptidase family protein | High confidence | + | - | - |
| WP_025096993.1 | adenine phosphoribosyl transferase | " | + | - | - |
| WP_047835678.1 | Probable thiol peroxidase | " | + | - | - |
| WP_047837194.1 | Pseudouridine synthase | " | + | - | - |
| WP_047839063.1**^¥^** | Nitro reductase family protein | " | + | - | + |
| WP_017920688.1 | Tail assembly chaperone | " | + | - | - |
| WP_047835815.1 | Ribonuclease PH | " | + | - | - |
| WP_047836206.1 | Fels-2 prophage protein | " | + | - | - |
| WP_047836637.1 | Transcriptional regulator | " | + | - | - |
| WP_047836698.1 | Signal recognition particle receptor FtsY | " | + | - | - |
| WP_047836541.1 | folate-binding protein | " | + | - | - |
| WP_047837207.1 | Alpha/beta hydrolase | " | + | - | - |
| WP_047836275.1 | phenylacetate-CoA oxygenase | " | + | - | - |
| WP_047837624.1 | Lysine--tRNA ligase | " | + | - | - |
| WP_025097569.1 | Amylo-alpha-1,6-glucosidase | " | + | - | - |
| WP_047838208.1 | serine threonine kinase protein | " | + | - | - |
| WP_047838737.1 | Uncharacterized protein | " | + | - | - |
| WP_017918437.1 | selenide, water dikinase SelD | " | + | - | - |
| WP_047836349.1 | AMP nucleosidase | " | + | - | - |
| WP_047837252.1 | Glutamine--tRNA ligase | " | + | - | - |
| WP_025100717.1 | Peptidase S1 | " | + | - | - |
| WP_017921696.1 | ribonucleotide-diphosphate reductase | " | + | - | - |
| WP_047835596.1 | Mannose-1-phosphate guanylyl transferase | " | + | - | - |
| WP_047839786.1 | Uncharacterized protein | " | + | - | - |
| WP_047838418.1 | peptidase S49 family protein | " | + | - | - |
| WP_047836704.1 | Nucleotide-binding protein | " | + | - | - |
| WP_047836235.1 | Putative flagellin type IV | " | + | - | - |
| WP_017917954.1 | hypothetical protein | " | + | - | - |
| WP_013698736.1 | Cysteine desulfurase IscS | " | + | - | - |
| WP_013698774.1 | Amino-acid acetyltransferase | " | + | - | - |
| WP_036040945.1 | Aldehyde dehydrogenase family protein | " | + | - | - |
| WP_013698235.1 | nicotinate phosphoribosyl transferase | " | + | - | - |
| WP_046581414.1 | L-threonine dehydratase | " | + | - | - |
| WP_047836209.1 | Phage tail protein | " | + | - | - |
| WP_013696090.1 | DNA gyrase subunit B | " | + | - | - |
| WP_013690079.1 | alkyl hydroperoxide reductase | " | + | - | - |
| WP_036037572.1 | hypothetical protein | " | + | - | - |
| WP_025096473.1 | Acetolactate synthase | " | + | - | - |
| WP_013690596.1 | GntR-family transcriptional regulator | " | + | - | - |
| WP_036051305.1 | 23S rRNA methyltransferase | " | + | - | - |
| WP_025099020.1 | 30S ribosomal protein S1 | " | + | - | - |
| WP_013690030.1 | hypothetical protein | " | + | - | - |
| WP_013688930.1 | bacterial transferase hexapeptide family protein | " | + | - | - |
| WP_046580459.1 | Uncharacterized protein | " | + | - | - |
| WP_013690079.1 | alkyl hydroperoxide reductase | " | + | - | - |
| WP_013690596.1 | GntR-family transcriptional regulator | " | + | - | - |
| WP_036049580.1 | phosphoenolpyruvate carboxylase family protein | " | + | - | - |
| WP_036052401.1 | Cytidylate kinase | " | + | - | - |
| WP_060001795.1 | Threonine ammonia-lyase | " | + | - | - |
| WP_036038361.1**^¥^** | Acyl hydrolase | " | + | - | + |
| WP_013698774.1 | Amino-acid acetyltransferase | " | + | - | - |
| WP_047836582.1 | Polyamine-transporting ATPase | " | + | - | - |
| WP_013688930.1 | bacterial transferase hexapeptide family protein | " | + | - | - |
| WP_017920745.1 | Phage late control protein | " | + | - | - |
| WP_047837256.1 | Acid phosphatase | " | + | - | - |
| WP_047838951.1 | putative endo-beta-1,3-glucanase | " | + | - | - |
| WP_047836937.1 | Sensor histidine kinase | " | + | - | - |
| WP_047838854.1 | Uncharacterized protein | " | + | - | - |
| WP_047839272.1 | MFS transporter | Low confidence | + | - | - |
| WP_047836491.1 | Probable potassium transport system protein kup | " | + | - | - |
| WP_047836177.1 | F0F1 ATP synthase subunit A | " | + | - | - |
| WP_047837742.1 | phosphoglycolate phosphatase | " | + | - | - |
| WP_047837474.1 | 1-deoxy-D-xylulose-5-phosphate reductoisomerase | " | + | - | - |
| WP_047836519.1 | Aminotransferase DegT | " | + | - | - |
| WP_047836596.1**^¥^** | SAM-dependent methyltransferase | " | + | - | + |
| WP_047835749.1 | Octaprenyl diphosphate synthase | " | + | - | - |
| WP_047836483.1 | Cystathionine beta-lyase | " | + | - | - |
| WP_047836521.1 | Peroxiredoxin | " | + | - | - |
| WP_047837496.1 | DEAD/DEAH box helicase family protein | " | + | - | - |
| WP_044273657.1 | Transketolase | " | + | - | - |
| WP_017921844.1 | RNA helicase | " | + | - | - |
| WP_036035280.1 | Acetaldehyde dehydrogenase 2 | " | + | - | - |
| WP_036049931.1 | Acetyl-coenzyme A synthetase | " | + | - | - |
| WP_013697779.1 | ribosome-binding factor A | " | + | - | - |
| WP_036039774.1**^¥^** | Acyl-UDP-N-acetylglucosamine O-acyltransferase | " | + | - | + |
| WP_060004555.1 | Chemotaxis protein CheA | " | + | - | - |
| WP_013697779.1 | Ribosome-binding factor A | " | + | - | - |
| WP_060002131.1 | Hemolysin D | " | + | - | - |
| WP_013697084.1**^¥^** | Elongation factor 4 | " | + | - | + |

*****: Cut off value ≥0.8 represents high confidence and ≥0.5 represents low confidence.

#: Proteins were identified in culture supernatant using LC-MS/MS based proteome analysis. ‘+’ depicts presence while ‘-’ depicts the absence of the particular protein in extracellular milieu.

¥: These proteins are not considered as bonafide T3SS effector as they were found to be secreted in presence of CaCl_2_.

**Table S6. Effect of the endo-beta- 1, 3- glucanase mutant (NGJ105) and complement (NGJ106) strains of NGJ1 on secondary sclerotia production by *R. solani***

|  | **Replicate1*** | **Replicate2*** | **Replicate3*** |
| --- | --- | --- | --- |
| ***R. solani*** | 184 | 132 | 159 |
| ***R. solani* + NGJ1**  **(10^9^ cell/ml)** | 0 | 0 | 0 |
| ***R. solani* + NGJ1**  **(10^5^ cell/ml)** | 3 | 2 | 3 |
| ***R. solani* + NGJ105**  **(10^9^ cell/ml)** | 20 | 17 | 18 |
| ***R. solani* + NGJ105**  **(10^5^ cell/ml)** | 54 | 62 | 57 |
| ***R. solani* + NGJ106**  **(10^9^ cell/ml)** | 0 | 0 | 0 |
| ***R. solani* + NGJ106**  **(10^5^ cell/ml)** | 8 | 13 | 19 |

***:** Average number of sclerotia from three independent replicates.

**Table S7. Bacterial strains and plasmids used in this study**

| **Strains or plasmid** | **Relevant characteristics** | **Source/reference** |
| --- | --- | --- |
| ***E. coli* strains** |  |  |
| DH5α | F', endA1 hsdR17 (rk- mk+) supE44 thi-1 recA1 gyrA TelA1 cp8OdlacZAM15 A (lacZY A-argF) U169 | Lab collection |
| ***B. gladioli* strains** |  |  |
| NGJ-1 | Natural isolate | Lab collection |
| NGJ-101 | *Bg_9562*::pGD2 rif-2; Bg_9562^-^, Km^r^ derivative of NGJ-2 (Bg_9562 mutant strain) | Lab collection |
| NGJ-2 | *rif*-2, Rf^r^ derivative of NGJ-1 | Lab collection |
| NGJ-102 | NGJ-101/pGD3 (Bg_9562 compliment strain) | Lab collection |
| NGJ-12 | *HrcC*::pGD4 rif-2; T3S^-^, Km^r^ HR^-^ derivative of NGJ-2  (T3SS defective strain) | Lab collection |
| NGJ-104 | NGJ-100/pGD5 | Current study |
| NGJ-105 | *Glucanase*::pGD6 rif-2; Glucanase^-^, Km^r^ derivative of NGJ-2 | Current study |
| NGJ106 | NGJ-105/pGD7 | Current study |
| **Plasmids** |  |  |
| PK18mob | pUC18 derivative; Mob+ Tra– Km^r^ | Lab collection |
| pHM1 | Broad-host-range cosmid vector (13.3 kb); Sp^r^ | Lab collection |
| pGD5 | pHM1::*Bg_9562*(First 27bp deleted at 5’ terminal) | Current study |
| pGD6 | pK18mob::*endo-β-1, 3- glucanase*(partial gene) | Current study |
| pGD7 | pHM1:: *endo-β-1, 3- glucanase* (Full length) | Current study |

**Table S8. Primers used in this study**

| **Gene Names** | **Accession Number** | **Primer IDs** | **Primer sequence** | **Reference** |
| --- | --- | --- | --- | --- |
| *Bg_9562* | WP_047836209 | *Δ27*F | 5’ GGTACCATGACCAGCGACAACGCCGCG 3’ | Current study |
|  |  | *Δ27*R | 5’ AAGCTTCGCGCTCGGGGATTCCATGCT 3’ | Current study |
| *Bg_9562* | WP_047836209 | *9562*F | 5′ GGCGAGCAGACGATCAC 3′ | Current study |
|  |  | *9562*R | 5′ GACGTCGAGGTTGACGAG 3′ | Current study |
| *hrcC* | WP_013689095 | HrcCF | 5′ GTCGACCAACAAGACCAAGA 3′ | Current study |
|  |  | HrcCR | 5′ CGCCACCTTCACGAAGAA 3′ | Current study |
| *hrcW* | WP_047838857 | HrcWF | 5′ GCCAAGGAAGTGAAGGAGAAG 3′ | Current study |
|  |  | HrcWR | 5′ CGAACTGCTTGCACCAGA3′ | Current study |
| *hrcN* | WP_036054803 | HrcNF | 5’ GACCCTGATGGGCATGTT 3’ | Current study |
|  |  | HrcNR | 5’ CCAGGATCAGCTCGATGAAT 3’ | Current study |
| *hrcJ* | WP_047838862 | HrcJF | 5’ CTGGCGCAGACCATCAA 3’ | Current study |
|  |  | HrcJR | 5’ CATCACCAGCGTCTTGATCT 3’ | Current study |
| *hrpK* | WP_013689086 | HrpKF | 5’ AGTTCGTCAACGACAATCCC 3’ | Current study |
|  |  | HrpKR | 5’ CATGAAGCCCTGCATCTTCT 3’ | Current study |
| *Endo Beta- 1,3 glucanase* | WP_047838951 | BglucF | 5’ CGTCAACACCGCCGATAC 3’ | Current study |
|  |  | BglucR | 5’ CCACTGCACGTTGTACTTCT 3’ | Current study |
| Ribonuclease | WP_047835815 | RinuF | 5’ ATCTCGGTGGGCGTCTA 3’ | Current study |
|  |  | RinuR | 5’ ACCACGTTCATGTCGGTATC 3’ | Current study |
| *Phage late control* | WP_047836206 | PlcF | 5’ CAGGCAGACACTGTCCTATTC 3’ | Current study |
|  |  | PlcR | 5’ GCTCTTGATCAGCCATTCGAT 3’ | Current study |
| *Peptidase S49* | WP_047838418 | PepF | 5’ TGTGCTGATGGACGGTTT 3’ | Current study |
|  |  | PepR | 5’ GCGAGAAGGGATCGAAGAAA 3’ | Current study |
| *Hydrolase* | WP_047838932 | HydF | 5’ ACCGACATGTACGGCTACTA 3’ | Current study |
|  |  | HydR | 5’ CAGCAGCGCCTTGATGTA 3’ | Current study |
| *Acid-phosphatase* | WP_042285470 | ApF | 5’ GACATCCAGGGCTTGAAGAA 3’ | Current study |
|  |  | ApR | 5’ GATCTGCATCTGGTTCTGGTAG 3’ | Current study |
| *Sensor histidine kinase* | WP_047836937 | ShkF | 5’ CTGGAAACCGATGCCTATGT 3’ | Current study |
|  |  | ShkR | 5’ CCGAGCACCTGGTAGTAGA 3’ | Current study |
| *Hypothetical protein* | WP_047838001 | HpF | 5’ GAAGGATCCGGATTGGTTCTAC 3’ | Current study |
|  |  | HpR | 5’ TTGGCTTGCGGCGAATA 3’ | Current study |
| *Endo Beta- 1,3 glucanase* | WP_047838951 | *Δ*GluF | 5’ GAATTCACGCTATGCGTTCCTGTTCA 3’ | Current study |
|  |  | *Δ*GluR | 5’ TCTAGAGTAGAGGTACGGCTTCTCGC 3’ | Current study |
|  |  | GlucfF | 5’AATTCAGGTACCATGTTGAAGCTTCGTCA3’ | Current study |
|  |  | GlucfR | 5’AATTCAGAATTCTCACGGATAACTGACCA3’ | Current study |

**References**

Bartra SS, Jackson MW, Ross JA, Plano G V. Calcium-regulated type III secretion of Yop proteins by an Escherichia coli hha mutant carrying a Yersinia pestis pCD1 virulence plasmid. Infect Immun. 2006;74:1381–6.

Dasgupta N, Ashare A, Hunninghake GW, Yahr TL. Transcriptional induction of the Pseudomonas aeruginosa type III secretion system by low Ca2+ and host cell contact proceeds through two distinct signaling pathways. Infect Immun. 2006;74:3334–41.

DeBord KL, Galanopoulos NS, Schneewind O. The ttsA gene is required for low-calcium-induced type III secretion of Yop proteins and virulence of Yersinia enterocolitica W22703. J Bacteriol. 2003; 185(12):3499-54.

Ferracci F, Schubot FD, Waugh DS, Plano G V. Selection and characterization of Yersinia pestis YopN mutants that constitutively block Yop secretion. Mol Microbiol. 2005;57:970–987.

Fishman MR, Zhang J, Bronstein PA, Stodghill P, Filiatrault MJ. Ca2+-induced two-component system CvsSR regulates the type III secretion system and the extracytoplasmic function sigma factor AlgU in Pseudomonas syringae pv. tomato DC3000. J Bacteriol. 2018; 20:00538-17.

Gode-Potratz CJ, Chodur DM, McCarter LL. Calcium and iron regulate swarming and type III secretion in vibrio parahaemolyticus. J Bacteriol. 2010;192:6025–38.

Horsman SR, Moore RA, Lewenza S. Calcium Chelation by Alginate Activates the Type III Secretion System in Mucoid Pseudomonas aeruginosa Biofilms. PLoS One. 2012;7(10):e46826.

Jiang GF, Jiang B Le, Yang M, Liu S, Liu J, Liang XX, et al. Establishment of an inducing medium for type III effector secretion in Xanthomonas campestris pv. campestris. Brazilian J Microbiol. 2013; 44(3):945-52.

Lee PC, Stopford CM, Svenson AG, Rietsch A. Control of effector export by the Pseudomonas aeruginosa type III secretion proteins PcrG and PcrV. Mol Microbiol. 2010;75:924–41.

Liu AC, Thomas NA. Transcriptional profiling of Vibrio parahaemolyticus exsA reveals a complex activation network for type III secretion. Front Microbiol. 2015; 6:1089.

Straley SC, Plano G V., Skrzypek E, Haddix PL, Fields KA. Regulation by Ca2+ in the Yersinia low‐Ca2+ response. Molecular Microbiology. 1993; 8(6):1005-10.

Vallis AJ, Yahr TL, Barbieri JT, Frank DW. Regulation of ExoS production and secretion by Pseudomonas aeruginosa in response to tissue culture conditions. Infect Immun. 1999; 62(2):914-20.
